# Supplementary material for: Structural properties of a haemophore facilitate targeted elimination of the pathogen Porphyromonas gingivalis
Source: Nat Commun. 2018 Oct 5;9:4097. doi: 10.1038/s41467-018-06470-0 (PMC6173696; doi:10.1038/s41467-018-06470-0)
Supplement: Supplementary file 1 — Supplementary Information [file 41467_2018_6470_MOESM1_ESM.pdf]

Supplementary Information

Structural properties of a haemophore facilitate targeted elimination of the pathogen *Porphyromonas gingivalis*

Gao et al.

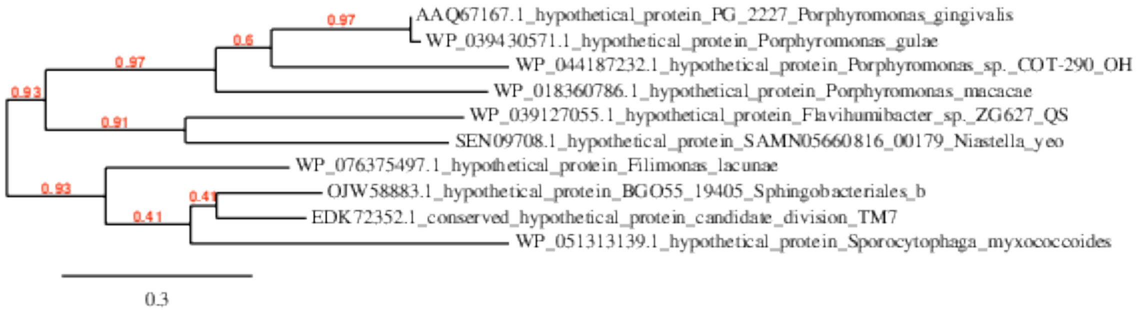


```
Porphyromonas macacae          MKTCRLIVLLMGIALMAAMTAKAQDSREYKKIMNAKVEALEVAP-LSGFLDLAADFERIA   59
Porphyromonas sp  COT290       -----------------------AQSSAYQRAMTERTAKLDSAS-IEVLQSLAADFDRFT   36
Porphyromonas gingivalis       MKTFKRIALLLV-AGFAGLCATSAQGTAYAEVMNRKVAALDSVP-PTEYATLAADFSRIA   58
Porphyromonas gulae            MKTFKRIALLLV-AGFAGLCATYAQGTAYAEVMNRKVAALDSVP-PTEYATLAADFSRIA   58
Sporocytophaga myxococcoides   ---------------------SNAQDDKFVKAMQKNLKALDSAKTTAEYIAISNSFERIG   39
Filimonas lacunae              --------AFFVVAAICSVMGVQAQSDKYTQAMERTLTLLDSAKTTEQLQTASAAFERIG   52
Sphingobacteriales bacterium   -------------------IAASAQSDKYTQMMQKNIALLDSAKSIDDLQSLASTFDRIG   41
Candidate division TM7         -----KKYLFLLLAAVGLQFAANSQSDKFISAMKTNLALFDSAKTVEDFNKMANTFERIG   55
Flavihumibacter sp ZG627       -----------------------AQSDKYKTAMVQQIAKLDGAMQNGGFTDLANSFERIG   37
Niastella yeongjuensis         -----MKQLFFILVSIVIVSQGIAQSEKFTKAMTTSIATFDSAKTADDMLAASAAFERIG   55
Consensus                                              :.  :   *      :: .         :  *.*:

Porphyromonas macacae          ENPESDWIAAYYSAYCRIVYALRTPA--KADELCEEADLMLKKSEEKNG---DHSEIACL   114
Porphyromonas sp  OH290        LMTGADWTAPYYSAYCRAIQAFADSE--AADRLAEQAEQYLEKATELGG---DASEIACL   91
Porphyromonas gingivalis       AVEGSDWMAAYYTAYCRIIPAFGNPS--EADRLCEEAESMLSKAESLGG---DLSEIACL   113
Porphyromonas gulae            AVEGSDWMAAYYAAYCRIIPAFGNPS--EADRLCAEAESMLSKAESLGG---DLSEIACL   113
Sporocytophaga myxococcoides   DAEKTSWLPYYYAALALITPAWNDKA-INADENSVKVKALIDKASKIED----NVEIYGL   94
Filimonas lacunae              DAEKTQWTPYYYAALAQVRIGFSDQK-ADKDAIATKAGTILSKGEAIEN----TADLCTI   107
Sphingobacteriales bacterium   DAEKTKWLPYYYAALAQTWVGWNPTV-QDKDANSAKINAYLAKAEALEK----NSELYAV   96
Candidate division TM7         DAEKTEWLPYYYAALSLNTAGWMPDL-KDKDANSERVNAFCDKAEALAKSNTDKSEIQAV   114
Flavihumibacter sp ZG627       DAEKEQWLPYYYAAYCQVMNGFMEKDKSKVDPLADKAEALITKAETIAG--APNSETEVI   95
Niastella yeongjuensis         DAEKTQWLPYYYASLSQIIYAFMKNDMSQADAYANKADDLLKKADALQ---PKNSEVSCI   112
Consensus                           .*   **:: .    .         *  . .      *.           :   :

Porphyromonas macacae          RNMSATARMMVDPQSRWERWGAEAEKQLQTAMAINPVNPRAFLLKAQSLMYTPAQFGGGI   174
Porphyromonas sp  OH290        RSMLFAARLQVNPQTRWQIYGPESAKQLQLAQEANPNNPRVYLLQAQSVAYTPAAYGGGK   151
Porphyromonas gingivalis       RSMAASARLLVNPQERWQTYGAESSRQLAVALEANPANPRAYFLQAQSLLYTPAQFGGGK   173
Porphyromonas gulae            RSMAASARLLVNPQERWQTYGVESSRQLAVALEANPANPRAYFLQAQSLLYTPAQFGGGK   173
Sporocytophaga myxococcoides   RNMIATQQMMIDPSTRWVNYGVDASTALKKGLAMDPENPRLNFLKGSSIFYTPTQFGGGK   154
Filimonas lacunae              RNMAATVQMLVDPMSRWQTYGAQAATALQNAIKLDPNNPRIYYLQGMSIFGTPVQFGGGK   167
Sphingobacteriales bacterium   ENMSATQQMLVDPQTRWATYGKTAGEALQKGLQADPNNPRLYYLQGMSLFGTPTQFGGGK   156
Candidate division TM7         RNMAATQQMLVDPQSRWASYGKTAGEALQKGMELNPNNPRLYYLQGMGLFGTPTQFGGGK   174
Flavihumibacter sp ZG627       RSMIASSRMMVDPQSRWMQYGQVSSGHMEKAKQQDPANPRPVYLEAQSKFYTPEQFGGGK   155
Niastella yeongjuensis         KSMIATLHMLVNPQNRWQEYGPVIQTELDNAKKQDPANPRPYFLQGQNLRNTPEQFGGGC   172
Consensus                      ..*  : :: ::*  **  :*      :  .   :* ***   *:. .   **  :***

Porphyromonas macacae          DKALPYMKKCLELFGQEESVSGYVPHWGAEMAKKLF---------   210
Porphyromonas sp  OH290        DKALPSITKALELYTKQSPEPAYAPHWGEEQAKALYHFCTAE---   193
Porphyromonas gingivalis       DKALPFAEKSVSCYAAATVSPAYAPHWGEQQARQLLMLCKAETQE   218
Porphyromonas gulae            DKALPFAEKSVSCYAAATVSPAYAPHWGEQQARQLLMLCKAESQE   218
Sporocytophaga myxococcoides   DKAKPVLQKAVELYSKEQPK-PLYPSWGKNSAEELLAQC------   192
Filimonas lacunae              DKAKPLFEKALSLSKTEQVK-PLHPHWGGKESEDMLAKCQ-----   206
Sphingobacteriales bacterium   DKAKPLFEKSVSLYKTAHPE-PLAPTWGQQQAEAMLEQC------   194
Candidate division TM7         DKAKPVFEKAVALYKEEKPA-QFYPHWGQKQAEDMVVQCQ-----   213
Flavihumibacter sp ZG627       AVAAPIFEKAIAMFDQFKPASEIHPSWGKSAAEYFLSQCK-----   195
Niastella yeongjuensis         TTAKPLLEEALKKYEAYKPESSIAPNWGKGQVEKILATCK-----   212
Consensus                        * *   :.:             * **    . :
```

Supplementary Figure 1. Alignment of the full-length HusA amino acid sequence from *P. gingivalis* W83 strain and bacterial homologues. The maximum likelihood phylogeny of HusA generated by MUSCLE using MABL website (http://www.phylogeny.fr). Regions of consensus domains and residues are presented. The secondary structure consensus is highlighted with red lines. Gene accession numbers and annotations are from Genbank database (http://www.ncbi.nlm.nih.gov/genbank/). *Porphyromonas gulae* (WP_039430571), *Porphyromonas macacae* (WP_018360786), *Porphyromonas sp.* COT-290 OH860 (WP_044187232), *Filimonas lacunae* (WP_076375497), *Sphingobacteriales bacterium* (OJW58883*), Flavihumibacter sp. ZG627*

(WP_039127055), *Niastella yeongjuensis* (SEN09708), *Sporocytophaga myxococcoides* (WP_051313139), candidate division TM7 genomosp.(EDK72352).

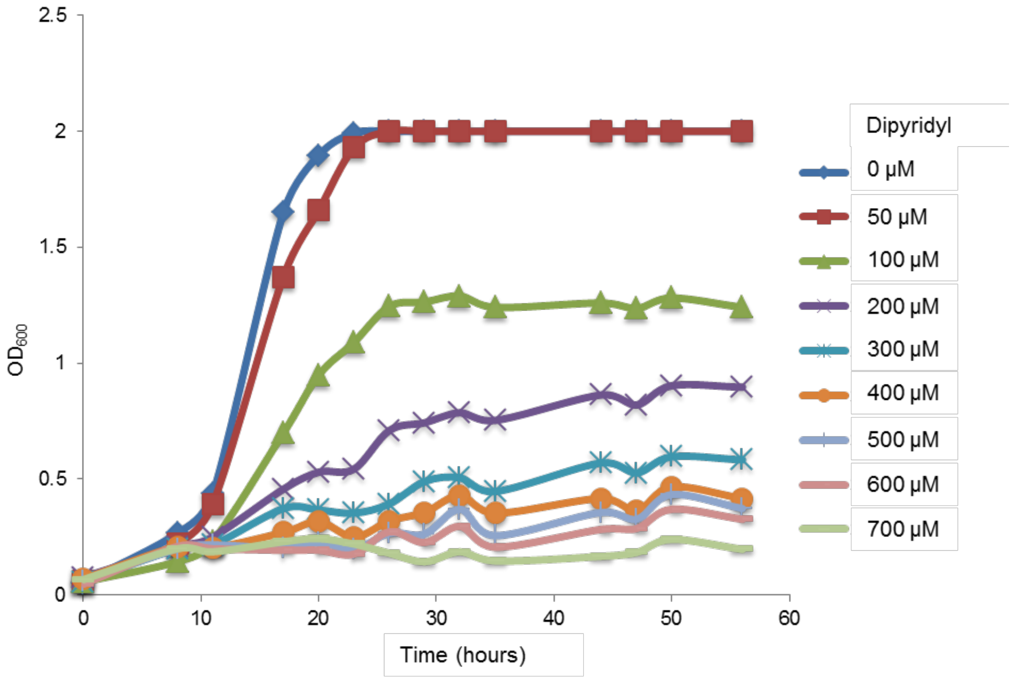

Supplementary Figure 2. Representative planktonic growth curves of *P. gingivalis* wild type W83 strain on 2,2′-dipyridyl titrations in eTSB medium. Dipyridyl is a known iron chelator, thus the total bacterial biomass decreased upon progressive titration of dipyridyl to sequester iron in the culture medium.

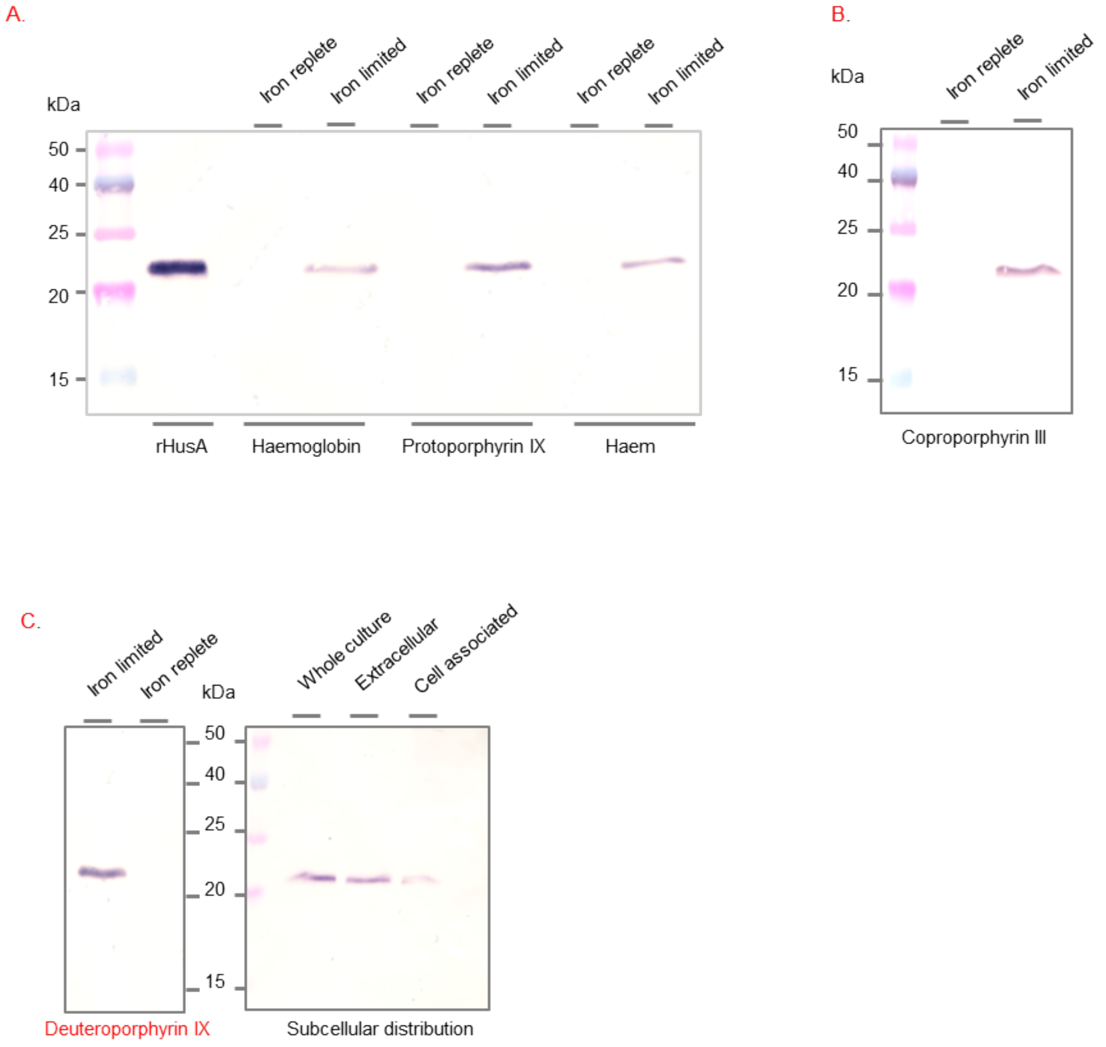

Supplementary Figure 3. Immunoblots of cell lysates or subcellular fractionations of *P. gingivalis* treated with (iron limited) or without (iron replete) dipyridyl and probed with antibody against HusA. Bacterial cells were harvested from growth media supplemented with: (a) haem, haemoglobin or protoporphyrin IX; (b) coproporphyrin III; (c) deuteroporphyrin IX. Samples were harvested and quantitatively normalized to the lowest biomass (OD_{600} of 0.5).

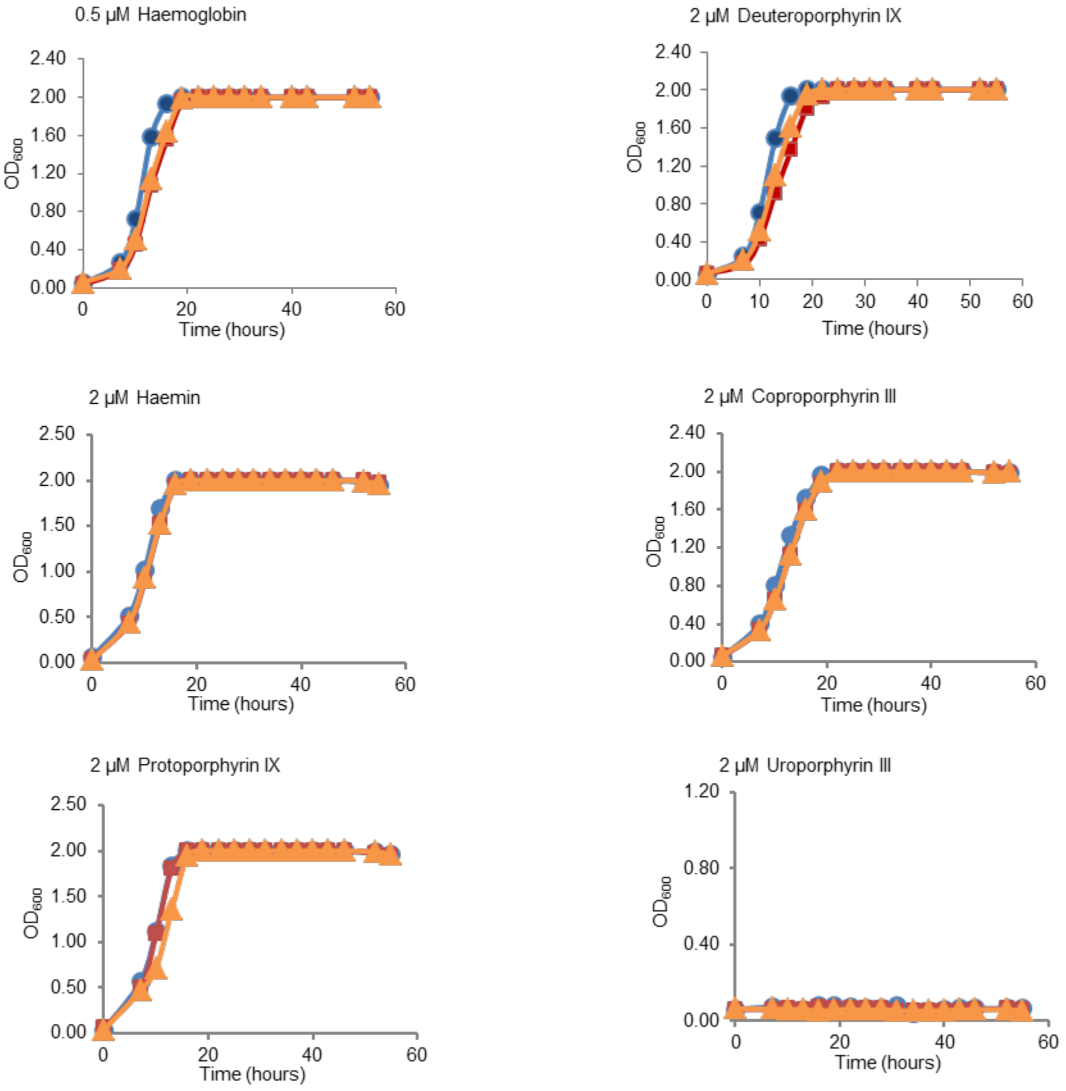

Supplementary Figure 4. Representative planktonic growth curves of *P. gingivalis* wild type W83 strain (blue circle), *husA* deleted mutant ΔhusA (burgundy square), and *husA* deleted control mutant ΔhusA^{+} (orange triangle) in the absence of iron chelator supplemented with different haem/porphyrin sources as follows: haemoglobin (0.5 µM), haemin (2 µM), protoporphyrin IX (2 µM), deuterophorphyrin IX (2 µM), coproporphyrin III (2 µM) and uroporphyrin III (2 µM).

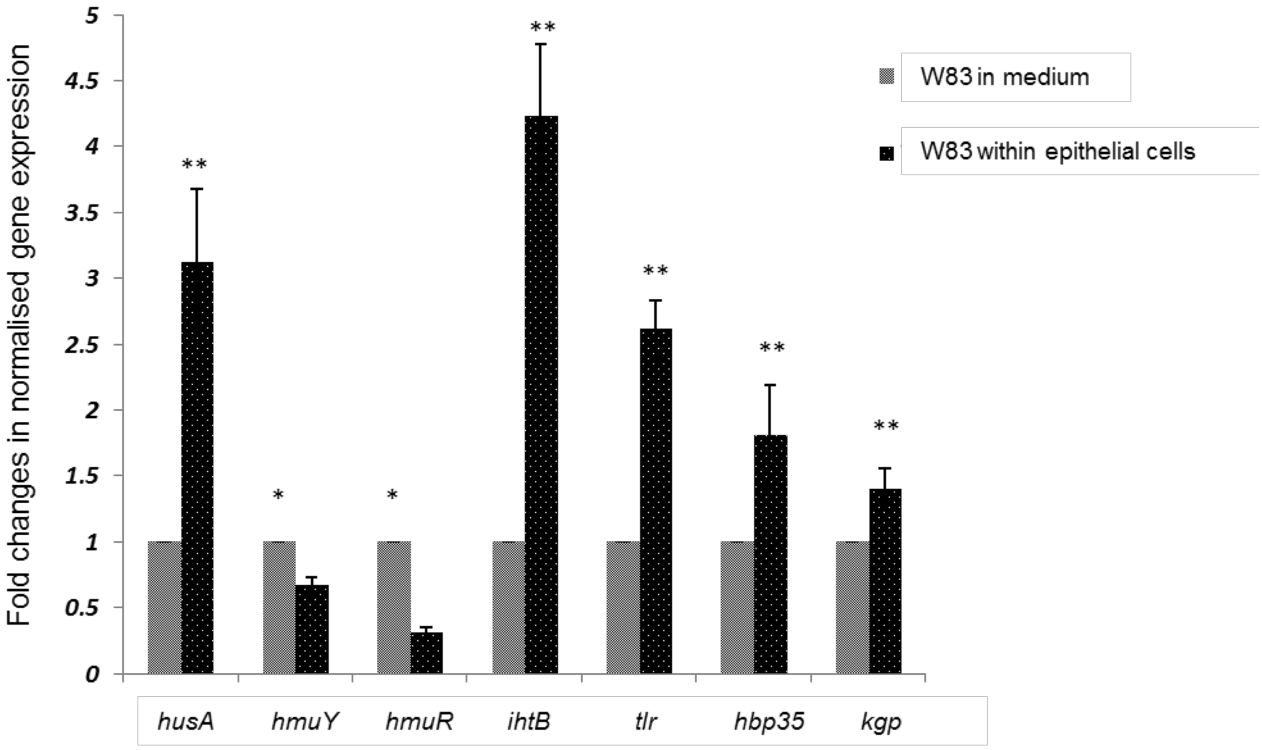

Supplementary Figure 5. Real-time qPCR analysis of known haem binding proteins expressed on the *P. gingivalis* surface, including HusA, HmuY, HmuR, Tlr, IhtB, and Kgp, post epithelial cell infection with wild-type W83 strain. Transcription levels were normalised against *P. gingivalis* 16s RNA and fold change was determined as described in Methods. *$p<0.01$, ** $p<0.001$.

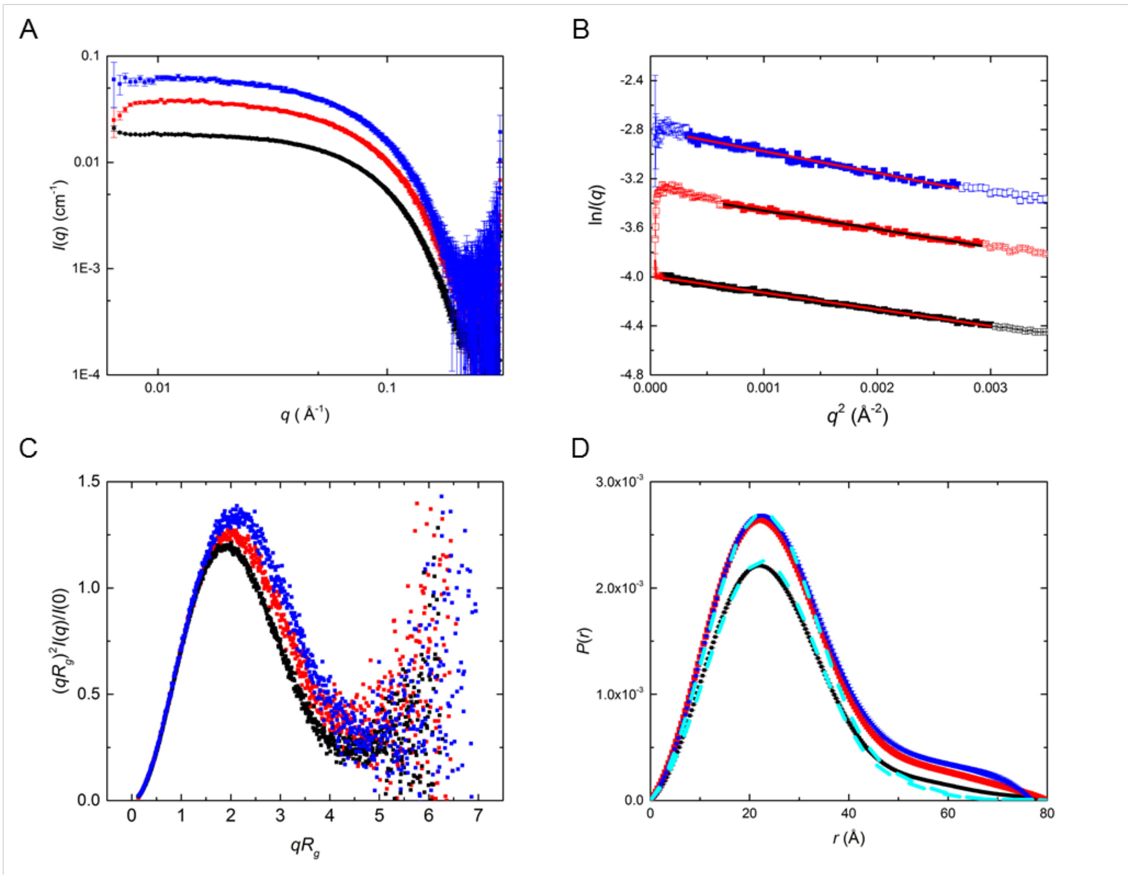

Supplementary Figure 6. SAXS results for the 1.5 mg/mL dataset for apo HusA in standard buffer (red) and the merged apo HusA measurements in reduced buffer (containing 2 mM TCEP; black), and for the single 3 mg/mL concentration measurement in the presence of a 1:1 molar equivalence of haemin in standard buffer (blue). For the apo HusA in reduced buffer, the data are scaled on merging to the corresponding lowest concentration data set in the series (1.5 mg/mL). (**a**) I(q) versus q as log-linear plots and (**b**) corresponding linear Guinier plots with the fitted region in filled symbols and open symbols indicating low-q data truncated to minimise the influence of the small amounts of aggregate present (as assessed by the molecular mass estimates) as well as data beyond the Guinier region ($qR_g < 1.2$). The reduced data set shows a turnover at low q, which can be attributed to parasitic scattering from the beam stop as there was no observed concentration dependence in these data and hence no inter-particle interference scattering that could potentially be the alternative cause for such a turnover. (**c**) Dimensionless Kratky plots for the data in (**a**) showing a small rise for qRg values > 4.5 indicating some limited flexibility in the protein under both buffer conditions and in the presence of haem (**d**) P(r) versus r profiles from the data in (**a**), q_{min} values for the transforms were the same as for the Guinier analyses, and q_{max} corresponded to the value where the SAXS profile flattens, which corresponds to the limit where the shape information dominates. Cyan dashed line is the P(r) profile calculated from the HusA NMR structure model profile, while red and black show the P(r) profiles of apo HusA in standard and reduced buffers, respectively, and blue shows the P(r) profile of HusA:haem at a molar ratio of 1:1. This sample was only measured in standard buffer.

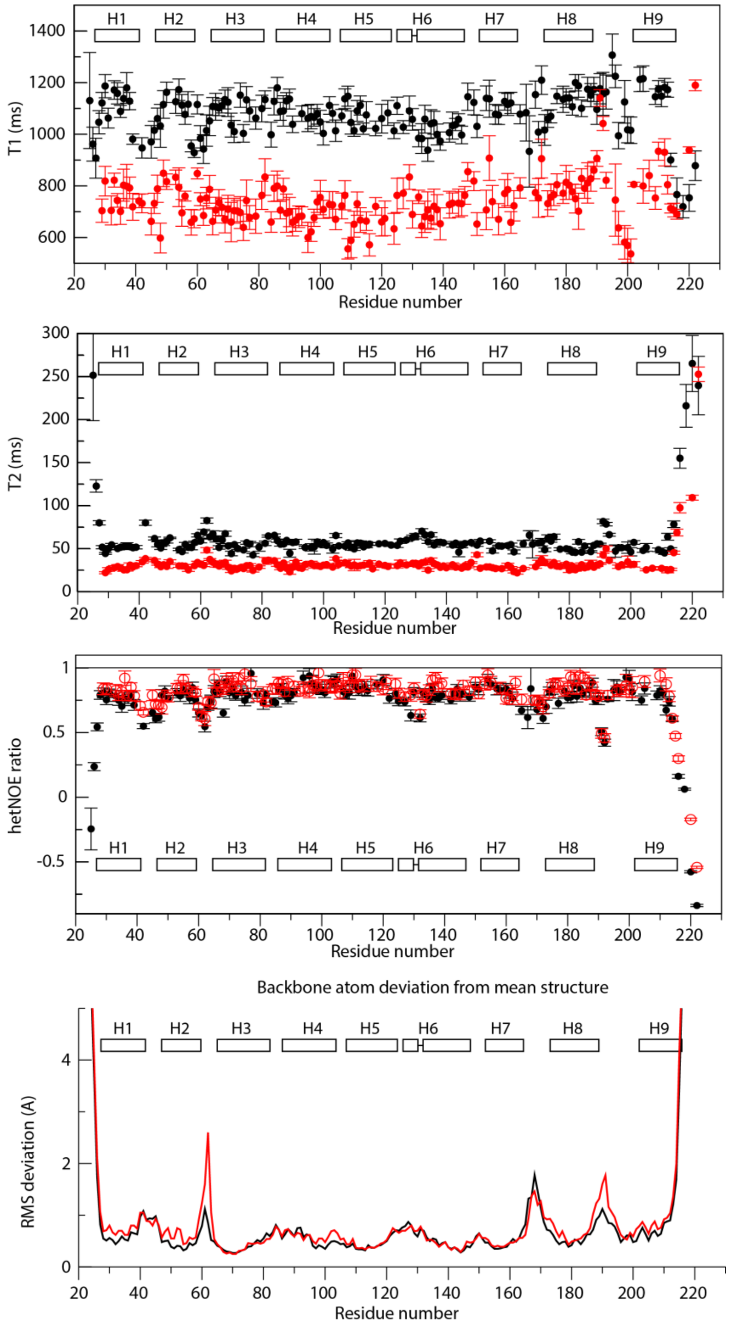

Supplementary Figure 7. HusA ^{15}N relaxation parameters and per-residue rms deviation in backbone coordinates. The T_1, T_2 and hetNOE parameters for each residue with a resolved signal in ^{15}N-HSQC at 298 K (black points, recorded at 600 MHz) or 308 K (red points, recorded at 800 MHz) are shown. The r.m.s. deviation in the position of backbone atoms across all 20 structures is plotted for NMR conformer set 1 (black line) and set 2 (red line). Large deviations in backbone r.m.s.d., T_2 and hetNOE are seen for the terminal residues (MGQGT and AETQELVPR). Internal residues in the ranges 60–63, 166–170 and 188–193 had r.m.s. deviations >1 Å. Internal residues 42, 47, 61, 62, 191, 192 had HetNOE values <0.7 in both 298/308 K data sets (with additional residues in ranges 42–47, 60–68 and 165–173 at 298 K). Internal residues 42, 62, 191, 192 had T_2 values >2 s.d. from the mean in both 298/308 K data sets (with additional residues 150, 132, 163 from one data set). Error bars are calculated as described in Supplementary Methods.

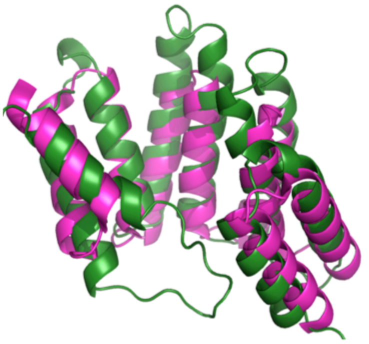

HusA (green); human O-GlcNAc transferase (pdb 5c1d; magenta)

```
TPR     ---φ--φA--φ----   ----A---φ--A- ---P
DSSP  LHHHHHHHHHHHHHHLLlllLHHHHHHHHHHHHhLLLLL
HusA  TAYAEVMNRKVAALDSVpptEYATLAADFSRIAaVEGSD
5c1da THADSLNNLANIKREQG---NIEEAVRLYRKAL-EVFPe
2pl2a -AEQNPLRLGVQLYALG---RYDAALTLFERAL-KENPQD

TPR   ---φ--φA--φ----         ----A---φ--A----P
DSSP  HHHHHHHHHHHHHHHllllllhhhHHHHHHHHHHHHHHHLLLH
HusA  WMAAYYTAYCRIIPAfgnpseadrLCEEAESMLSKAESLGGDL
5c1da AAAHSNLASVLQQQG---------KLQEALMHYKEAIRISPTF
2pl2a PEALYWLARTQLKLG---------LVNPALENGKTLVARTPRY

TPR   ---φ--φA--φ----           ----A---φ--A----P
DSSP  HHHHHHHHHHHHHHHhhllll-lhhhHHHHHHHHHHHHHHLLLLL
HusA  SEIACLRSMAASARLlvnpqe-rwqtYGAESSRQLAVALEANPAN
5c1da ADAYSNMGNTLKEMQ-----------DVQGALQCYTRAIQINPAF
2pl2a LGGYMVLSEAYVALYRQAEDReRGKGYLEQALSVLKDAERVNPRY

TPR   ---φ--φA--φ---     -----A---φ--A----P
DSSP  HHHHHHHHHHHHLLlhhhlLLHHHHHHHHHHHHHHHHL
HusA  PRAYFLQAQSLLYTpaqfgGGKDKALPFAEKSVSCYAA
5c1da ADAHSNLASIHKDS-----GNIPEAIASYRTALKLKPD
2pl2a APLHLQRGLVYALL-----GERDKAEASLKQALALED

DSSP  llllllllllllHHHHHHHHHHHHLLLL
HusA  ATVSPAYAPHWGEQQARQLLMLCKAETQ
5c1da ------------FPDAYCNLAHCLQivcdwtdydermkk
2pl2a
```

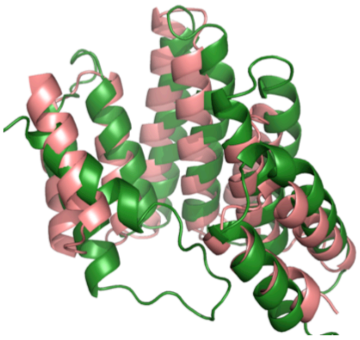

HusA (green); *Thermus thermophilus* TPR protein (pdb 2pl2; pink)

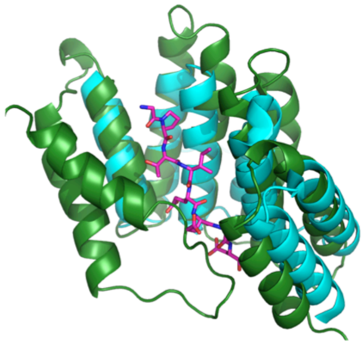

HusA (green); TPR domain of HOP, component of the Hsp70-Hsp90 chaperone complex (pdb 1elw; cyan) with peptide bound (magenta)

Supplementary Figure 8. HusA has homology to the tetratricopeptide repeat (TPR) proteins. Sequence alignment of the TPR-like amino acid motifs of HusA (residues 24–190, comprising α-helices 1–8) with TPR-repeat regions of human O-GlcNAc transferase 110 kDa subunit (pdb 5c1d) and hypothetical protein TTC0263 from *Thermus thermophilus* strain HB27 (pdb 2pl2). Secondary structure of HusA as calculated by dssp from the 3D coordinates with loop (L) or helical (H) regions as indicated. Each row shows alignment of the sequences to the 35-residue TPR repeat with positions of the repeat indicated as follows: (φ) hydrophobic residue; (A) Ala; (-) any residue. Superposition of the HusA structure (green shades) with structures pdb 5c1d (magenta), 2pl2 (pink), and 1elw (cyan) as determined by the program DALI [1]. For pdb 1elw (HOP), a bound hydrophilic peptide (magenta sticks) is shown. Other high-scoring hits from DALI search include the plant mitochondrial import receptor TOM-20 (1zu2) and various 14-3-3 proteins.

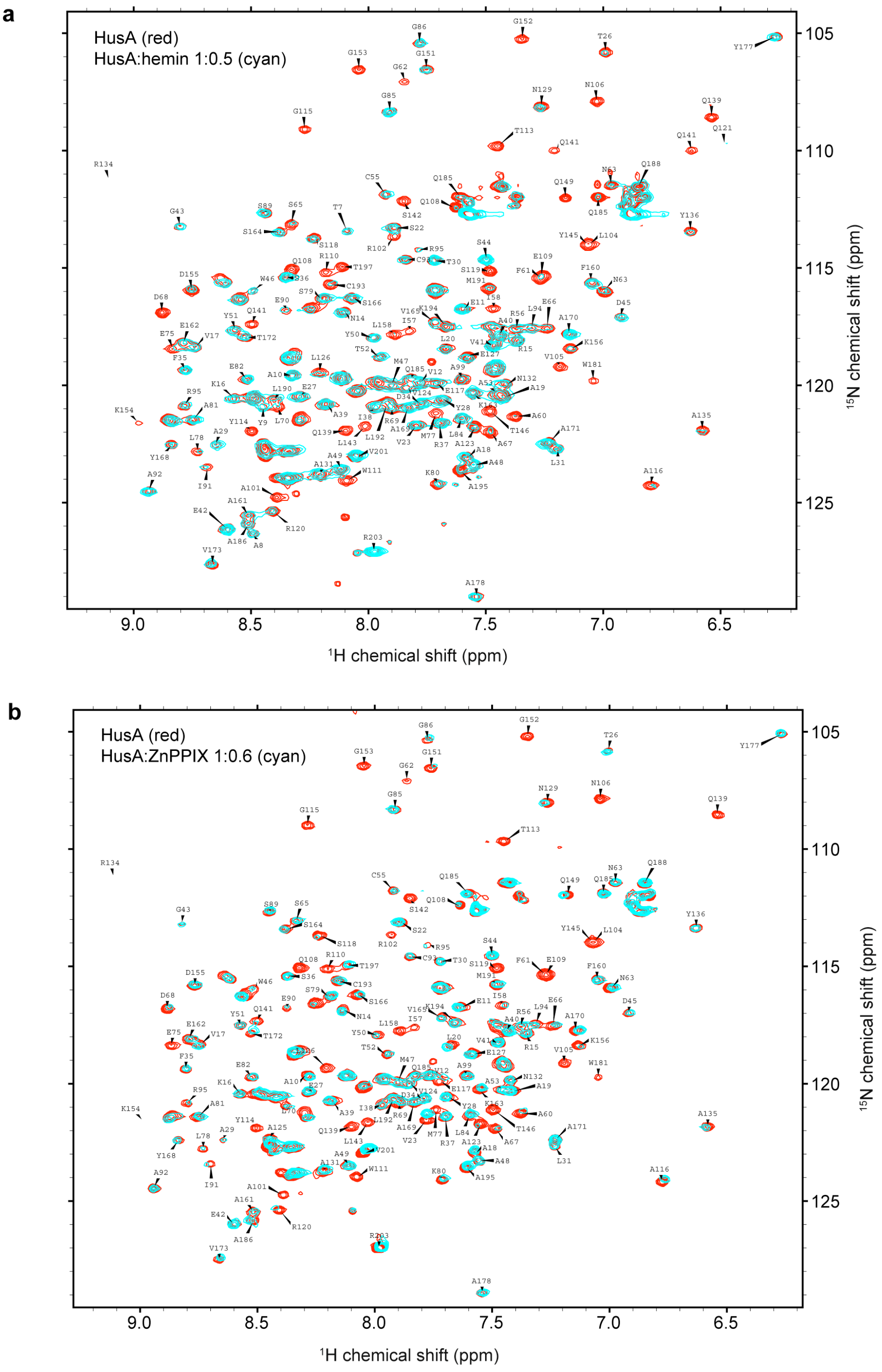

Supplementary Figure 9. Loss of a subset of HusA NMR signals upon addition of haemin or Zn-PPIX. ^{1}H-^{15}N-HSQC spectra of HusA in the absence and presence of (**a**) haemin and (**b**) Zn-PPIX. Similar sets of signals are lost to intermediate exchange in each case. [*U*- ^{15}N]rHusA at 0.4 mM in 0.1 M sodium phosphate buffer, pH 6.8, in the absence (red) or presence (cyan) of 0.5-molar equivalents of haemin, or 0.6-molar equivalents of Zn(II)PPIX.

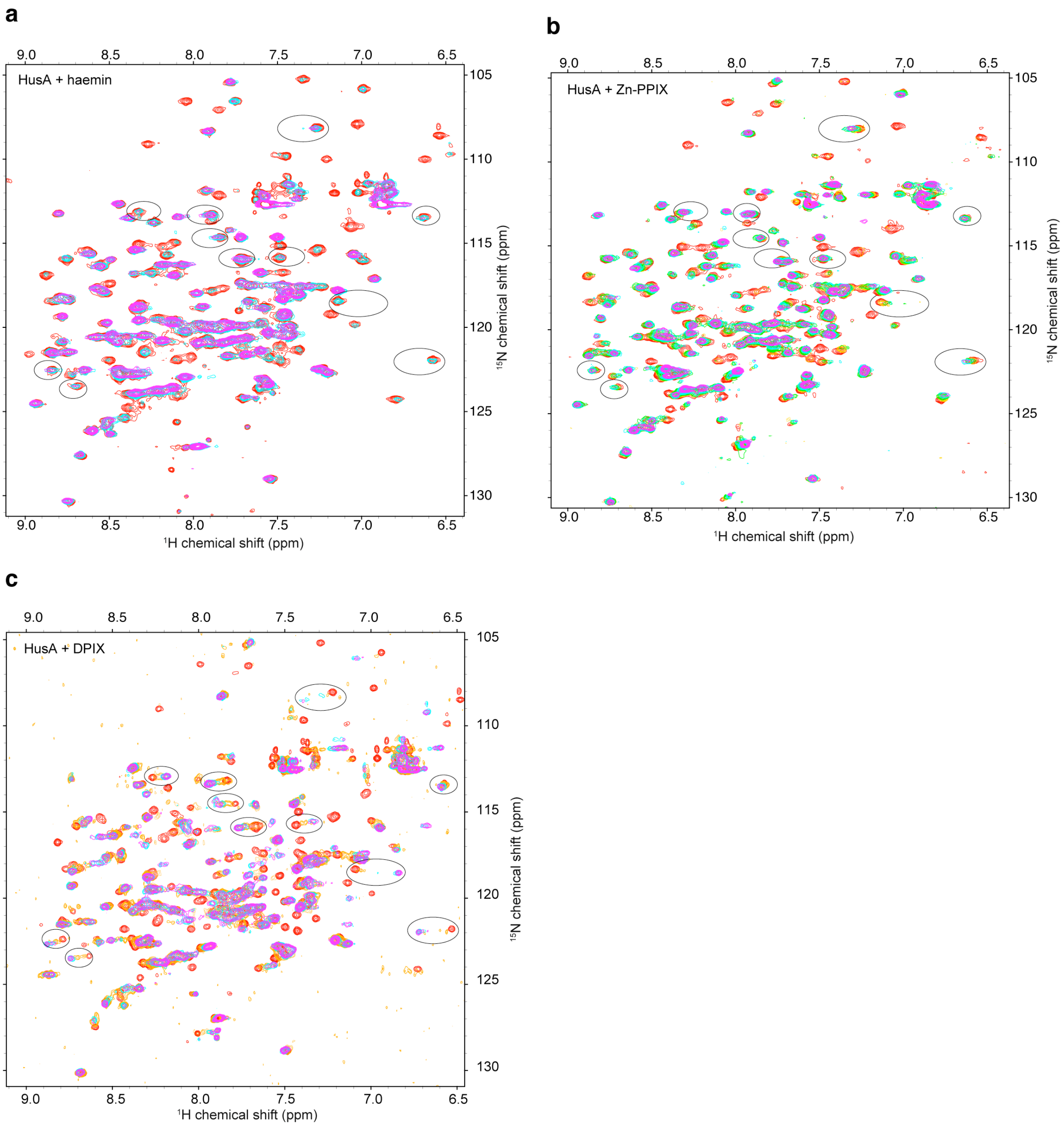

Supplementary Figure 10. NMR titration analysis of HusA with haemin, Zn-PPIX and DPIX at 298 K. ^{1}H-^{15}N-HSQC spectra recorded in the presence of (**a**) 0.5 molar equivalents (cyan), or 1 molar equivalent (magenta) of haemin; (**b**) 0.6 molar equivalents (yellow), 1 molar equivalent (green), 1.4 molar equivalents (cyan), or 2 molar equivalents (magenta) of ZnPPIX; (**c**) 1 molar equivalent (orange), 2 molar equivalents (cyan) or 3 molar equivalents (magenta) of DPIX. Similar sets of signals are lost to intermediate exchange in each case. In addition, selected signals appear to be in intermediate-to-fast exchange upon addition of DPIX (circled in each spectrum for comparison). Reaction mixture comprised 0.4 mM [*U*- ^{15}N]rHusA in 0.1 M sodium phosphate buffer, pH 6.8. Spectra were recorded at 298 K

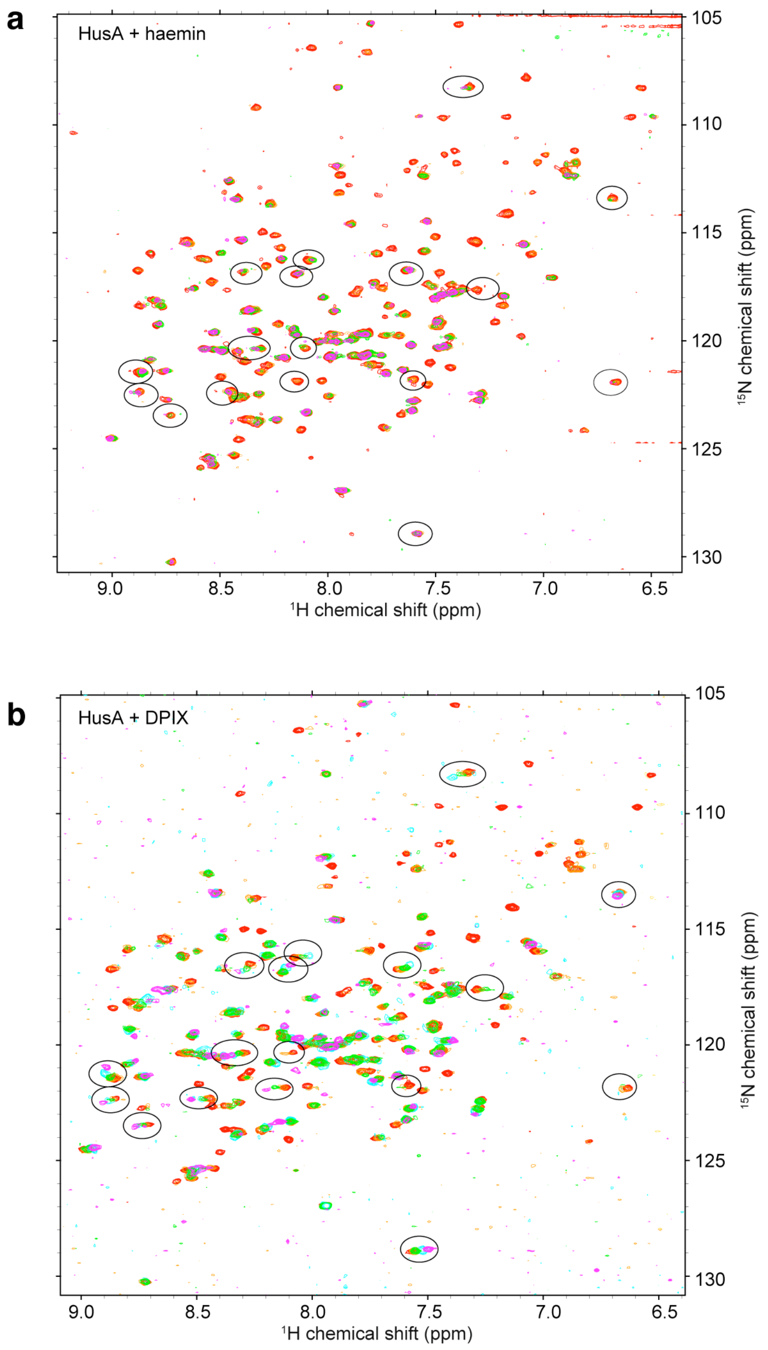

Supplementary Figure 11. NMR titration analysis of HusA with haemin or DPIX at 308 K. NMR titration analysis of HusA in the presence of (**a**) 0.3 molar equivalent (yellow), 0.7 molar equivalent (green), 1.4 molar equivalents (cyan), or 2 molar equivalents (magenta) of haemin; (**b**) 0.5 molar equivalent (yellow), 1 molar equivalent (green), 2 molar equivalents (cyan) or 3 molar equivalents (magenta) of DPIX. Selected signals that appear to be in intermediate-to-fast exchange in the case of DPIX are circled (see also comparison with Supplementary Figure 21b). Spectra recorded at 308 K

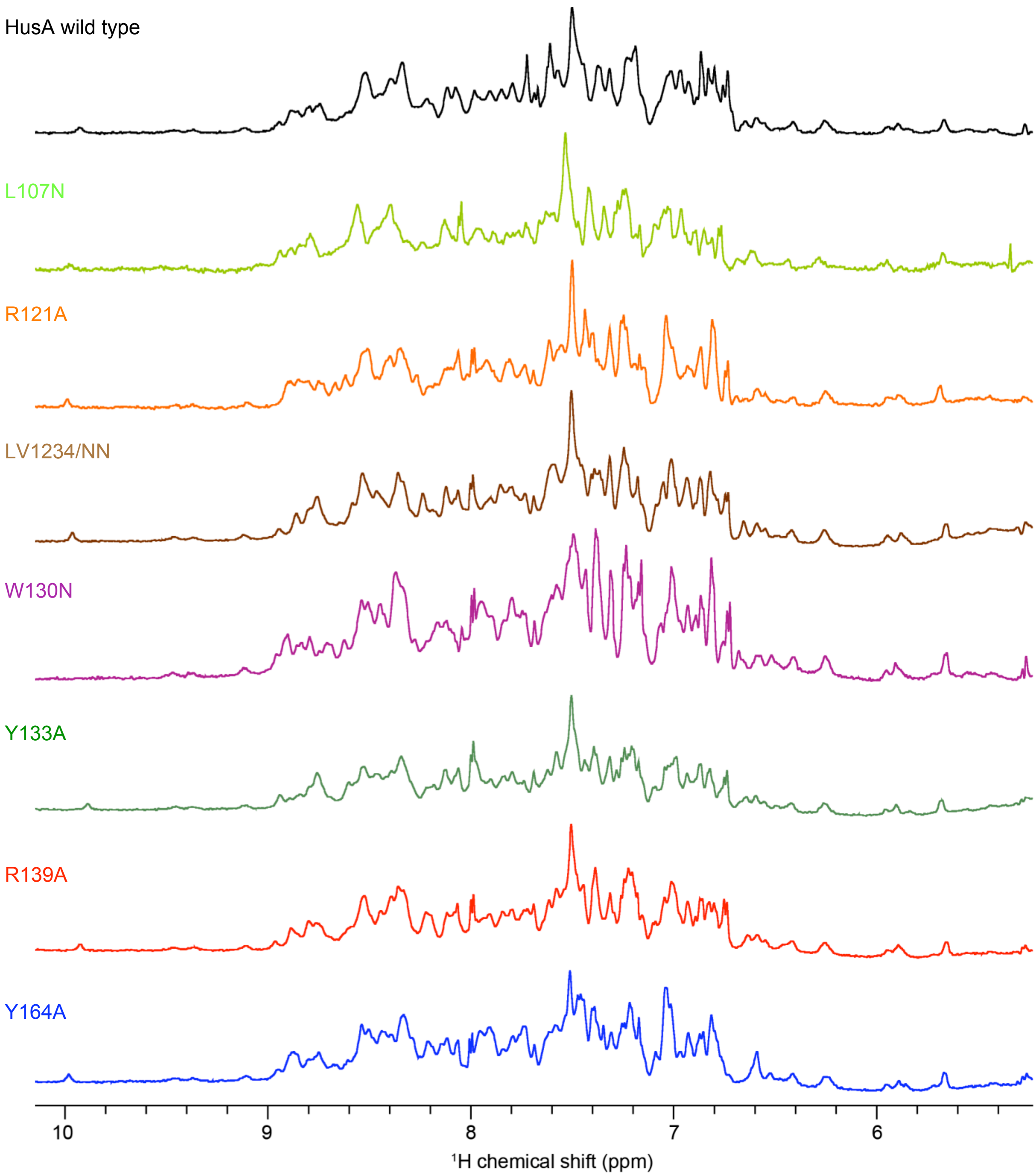

Supplementary Figure 12. Amide regions (5.5–10 ppm) of 1D ^{1}H NMR spectra of HusA and mutants show all proteins are well folded with sharp and dispersed peaks.

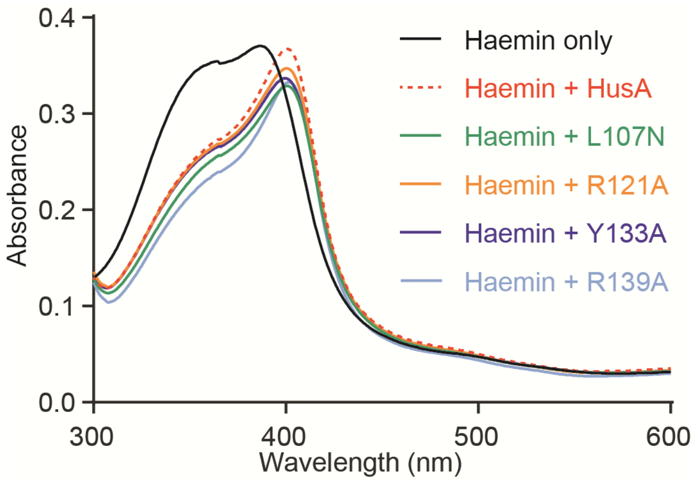

Supplementary Figure 13. Representative UV-vis spectra of 10 μM haemin, alone, and in the presence of 10 μM HusA and four mutants that displayed similar binding.

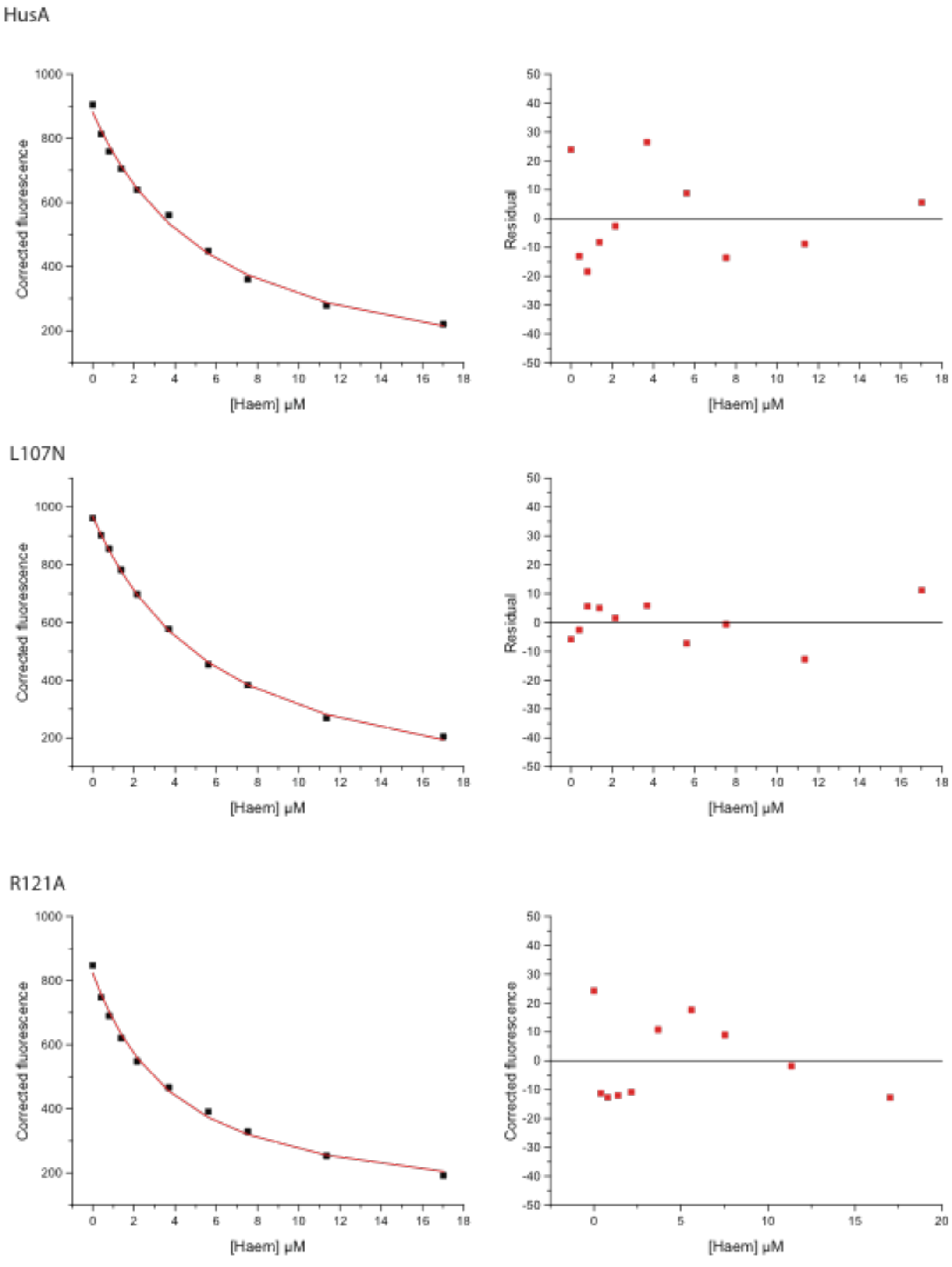

Supplementary Figure 14a. Titrations of HusA and HusA mutants L107N and R121A with haemin. Corrected fluorescence intensities (symbols) and fitted values (lines) for a 1:1 binding model are shown.

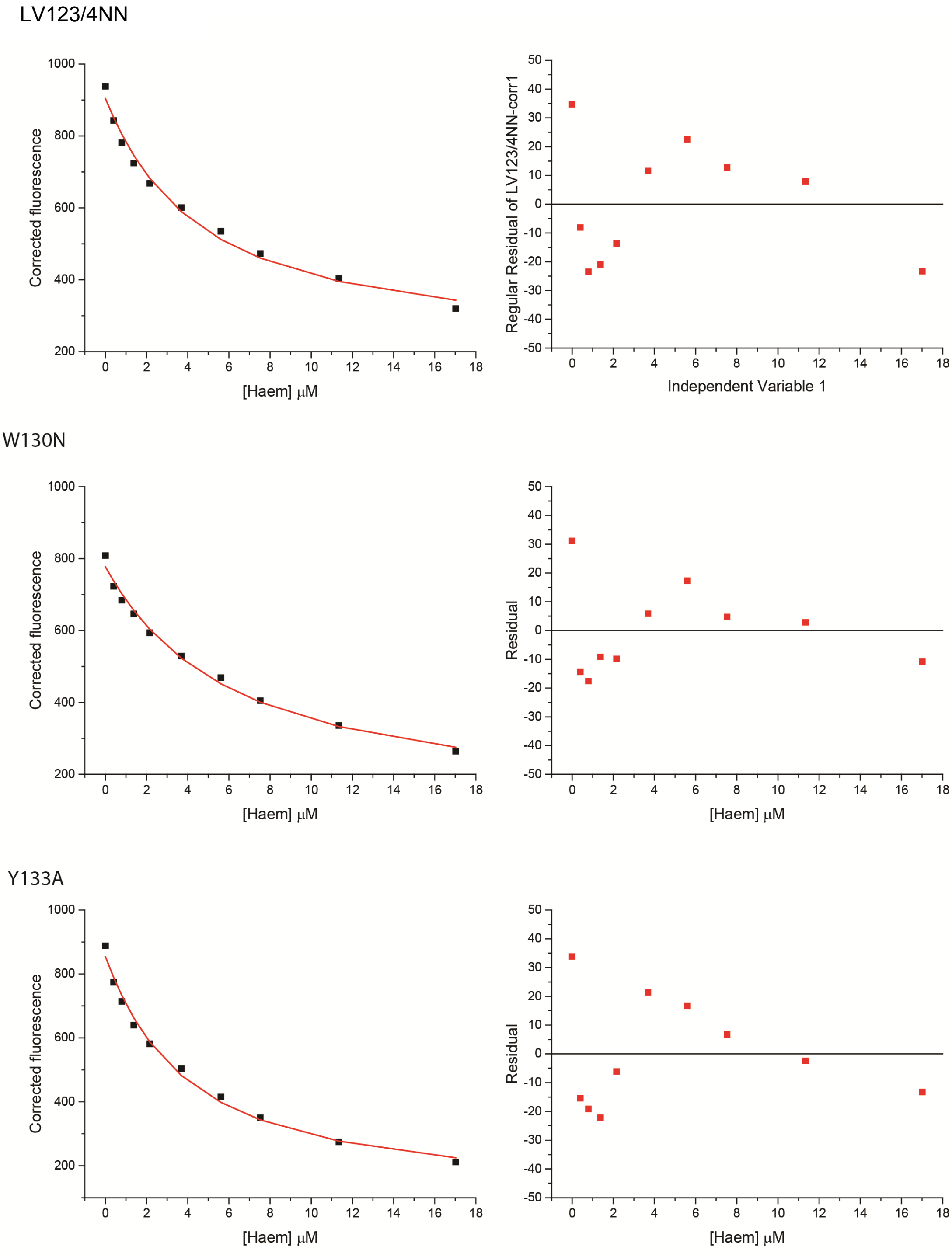

Supplementary Figure 14b. Titrations of HusA mutants LV123/4NN, W130N and Y133A with haemin. Corrected fluorescence intensities (symbols) and fitted values (lines) for a 1:1 binding model are shown.

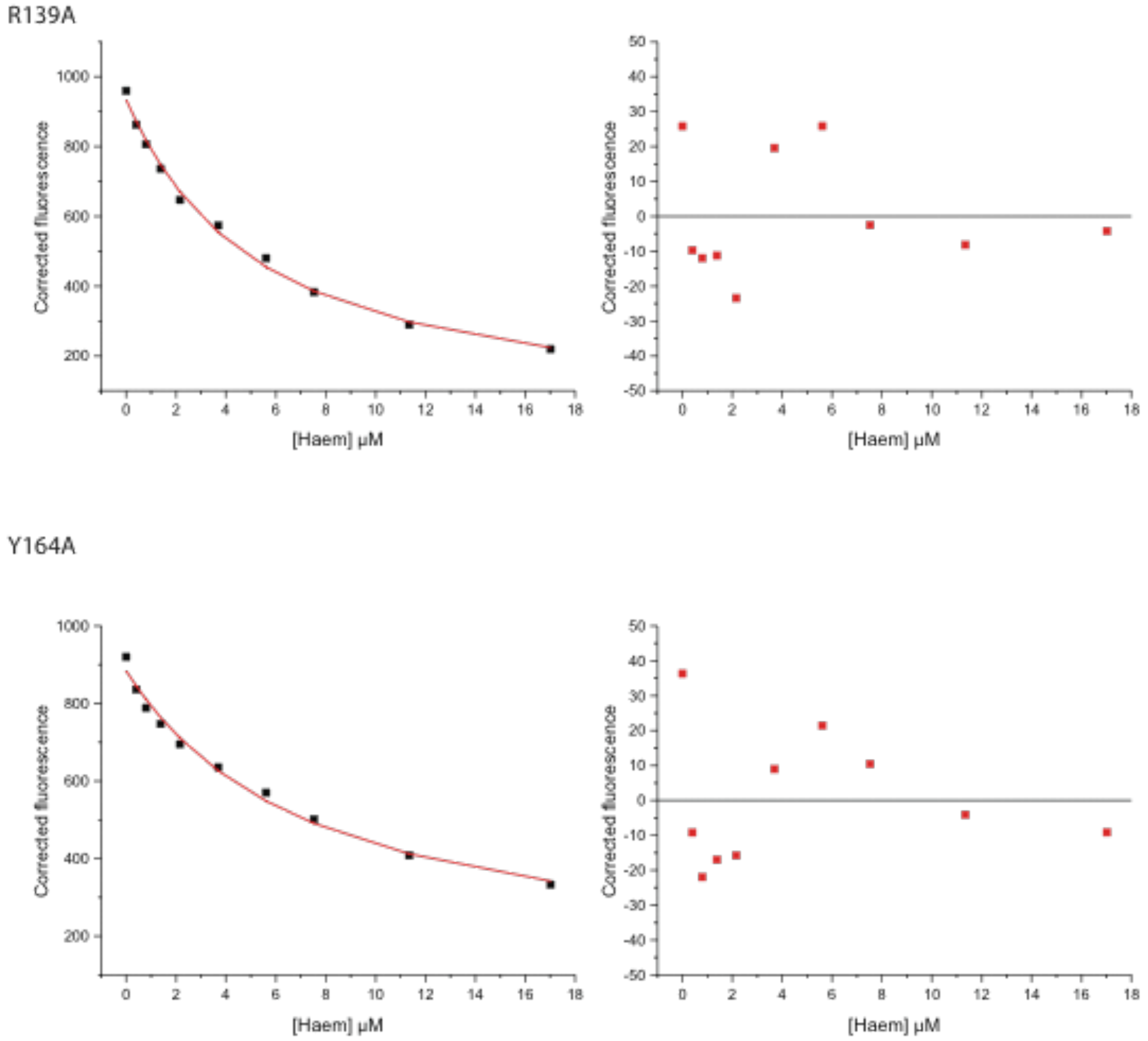

Supplementary Figure 14c. Titrations of HusA mutants R139A and Y164A with haemin. Corrected fluorescence intensities (symbols) and fitted values (lines) for a 1:1 binding model are shown.

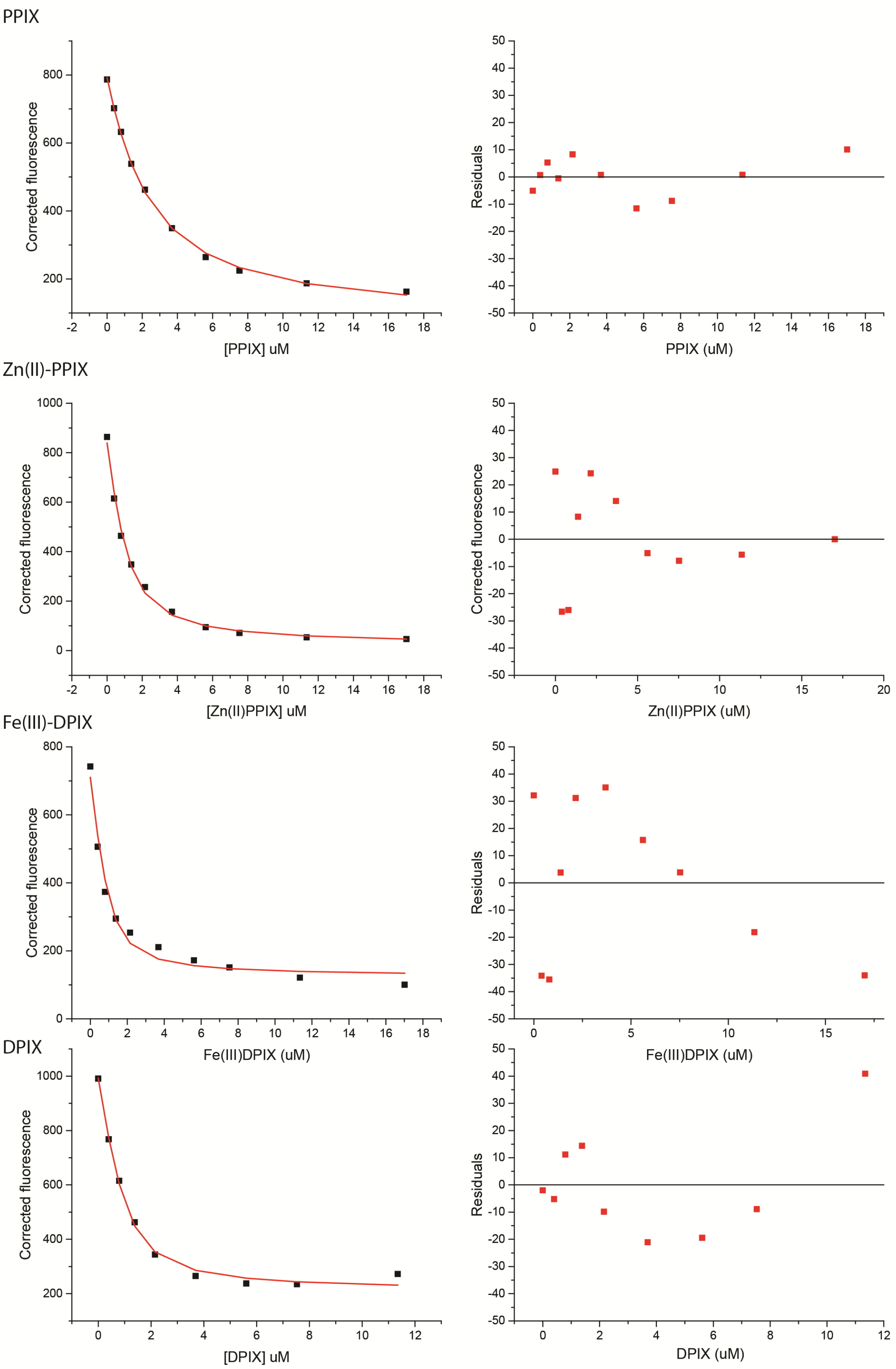

Supplementary Figure 14d. Titrations of PPIX, Zn(II)-PPIX, Fe(III)-DPIX and DPIX with HusA. Corrected fluorescence intensities (symbols) and fitted values (lines) for a 1:1 binding model are shown.

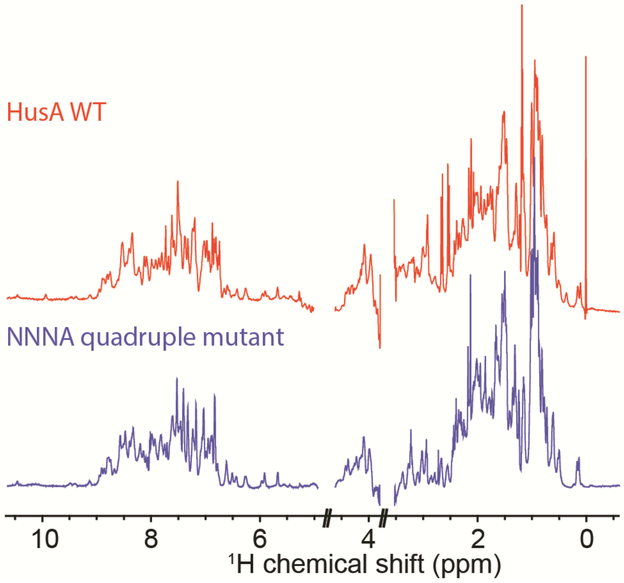

Supplementary Figure 14e. 1D ^{1}H NMR spectra of HusA and NNNA mutant.

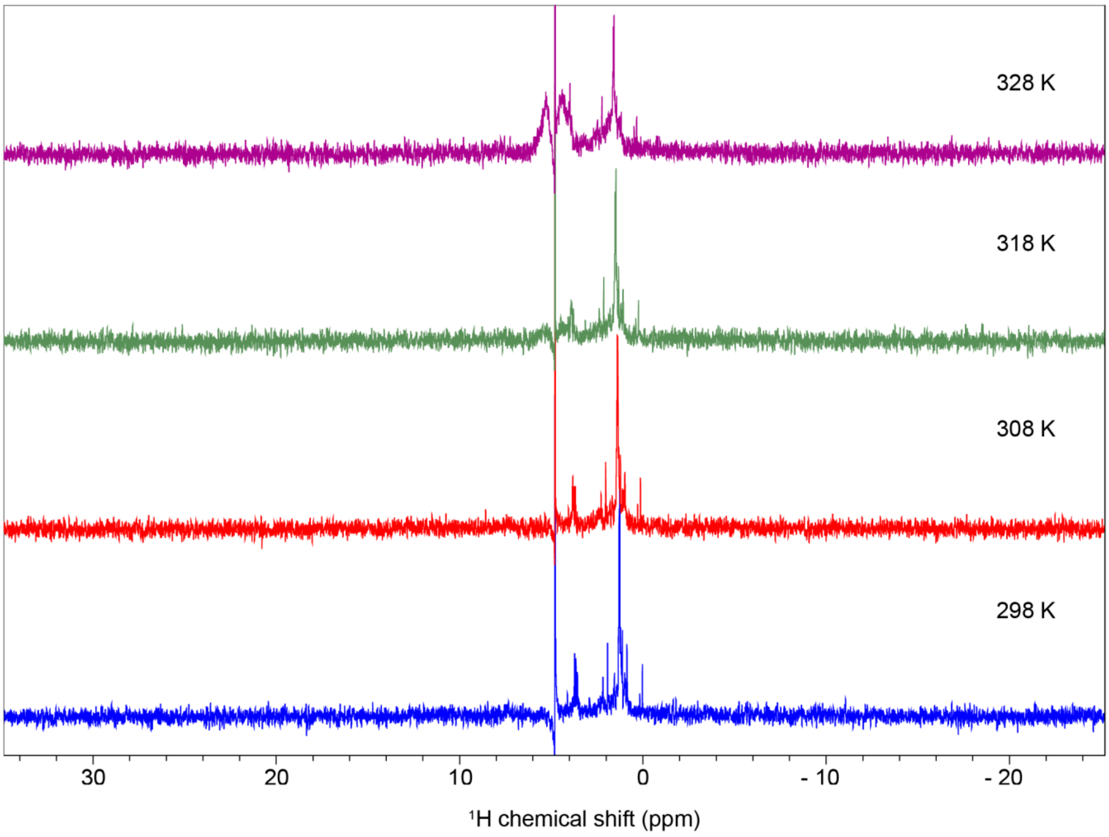

Supplementary Figure 15. 1D ^{1}H NMR spectra showing full spectral width (–25 to 35 ppm) of 240 ìM HusA:haem at 1:1. No haem hyperfine shift signals (i.e. beyond –2 to 10 ppm where protein and haem signals are normally found) can be seen at all temperatures tested from 298 K to 328 K.

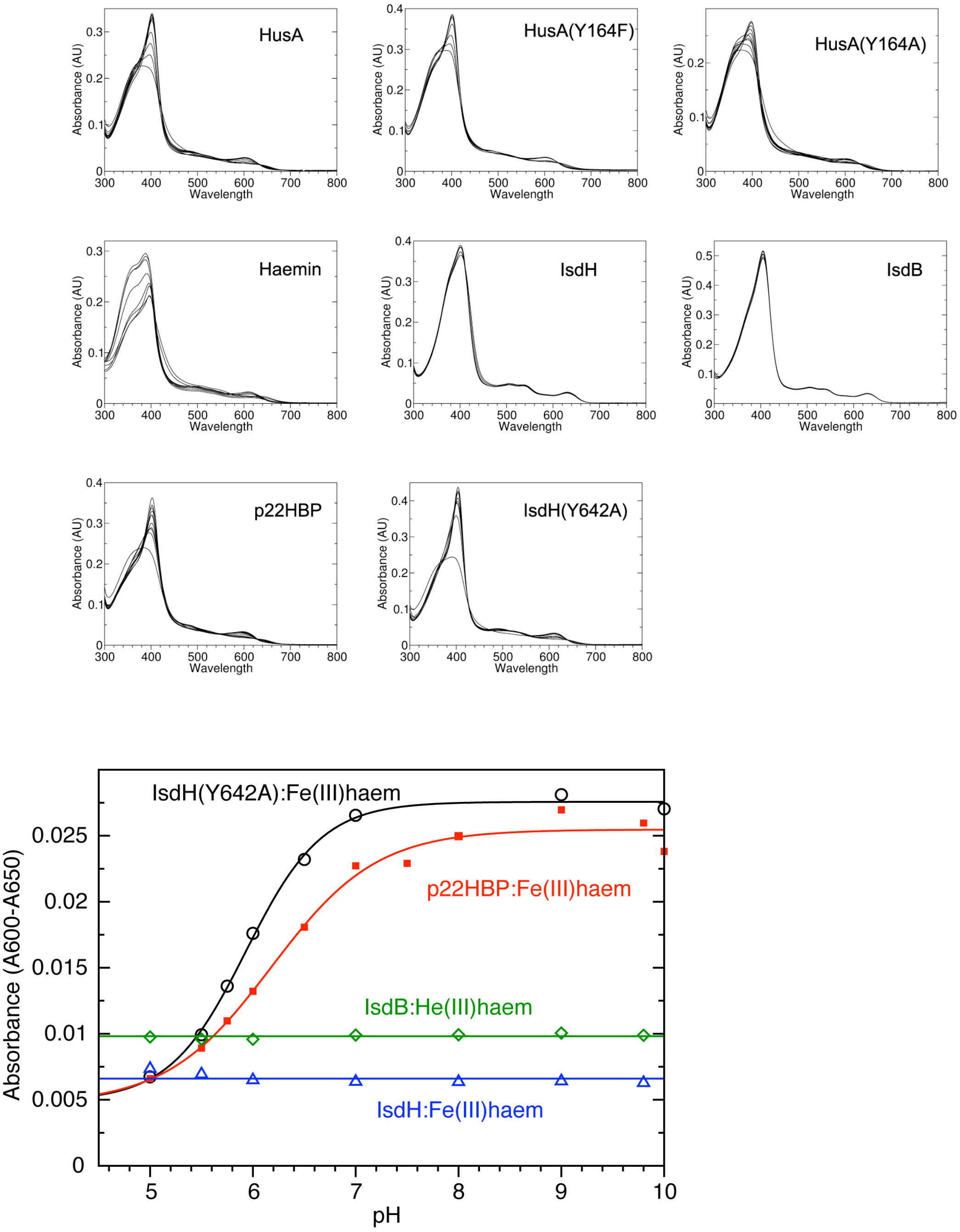

Supplementary Figure 16. Acid-alkaline transition in UV-vis spectra of various haem binding proteins. (Top) UV-visible spectra (units of wavelength are nm) used to generate the pH titration curves shown in Figure 2h and in the bottom panel. (Bottom) Acid-alkaline titration of 5 µM haemin in presence of 7 µM IsdH, 5 µM IsdB, 7 µM IsdH(Y642A) and 7 µM p22HBP. All proteins are in holo form. See Fig 2h legend for buffer conditions. Absorbance A600–A650 values are plotted except for IsdH and IsdB, for which A630–A600 is plotted (to avoid negative values).

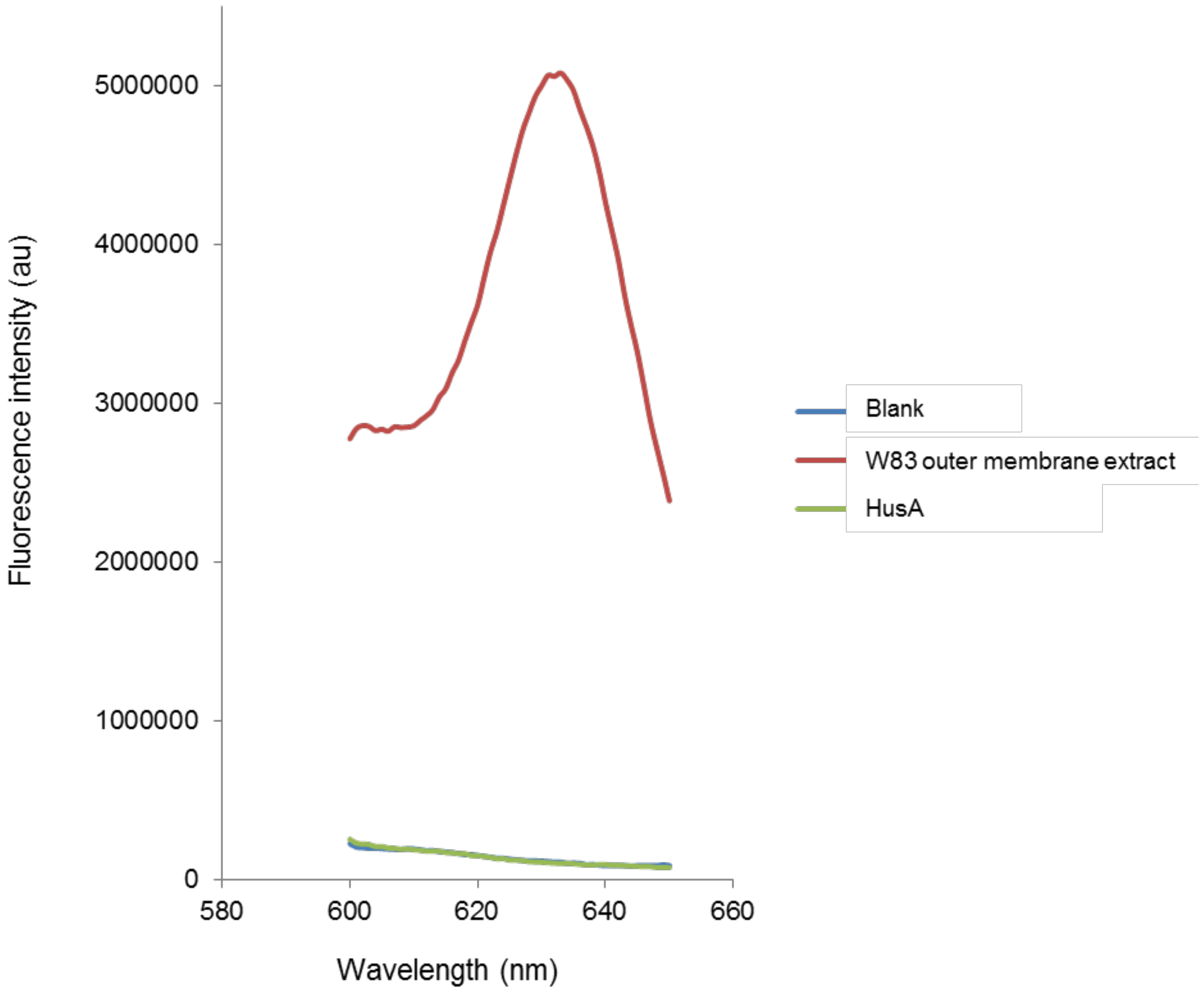

Supplementary Figure 17. Representative fluorescence spectra of reverse ferrochelatase activity assay. The fluorescence emission on treating haem solution with *P. gingivalis* outer membrane extract and recombinant HusA was recorded between 600 nm and 650 nm following excitation at 410 nm. The widths of emission and excitation slits were 5 nm. In the presence of reverse chelatase activity, the haemin was converted to protoporphyrin IX which gave rise to the emission peak at ~ 630-635 nm on excitation at 410 nm.

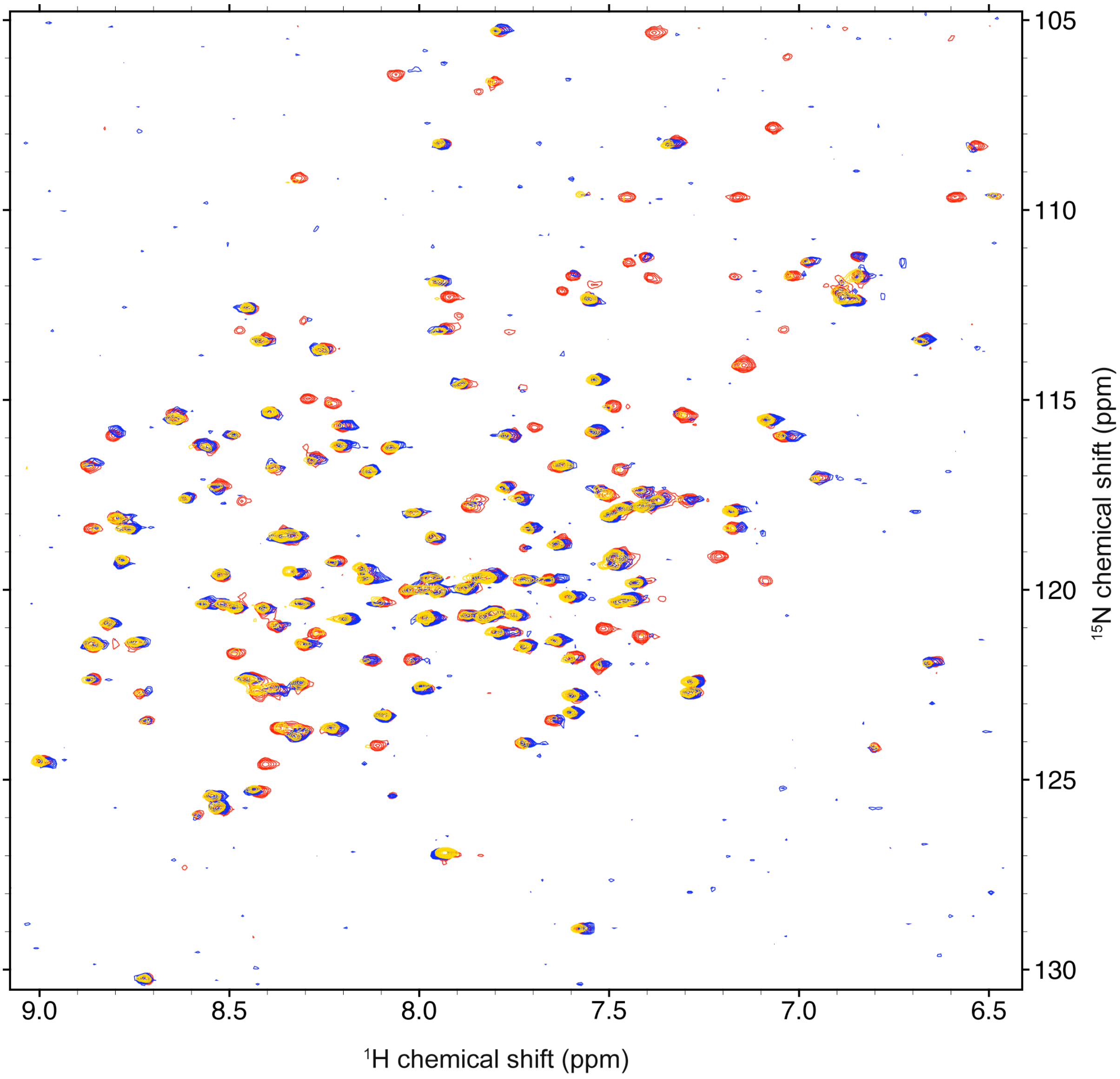

Supplementary Figure 18. Comparison of spectral changes for HusA upon addition of haemin or DPIX. ^{1}H-^{15}N-HSQC spectra of HusA with haemin or DPIX. [*U*- ^{13}C, ^{15}N]HusA (160 μM) in 10 mM sodium phosphate, 50 mM NaCl, 5% D_2O, pH 6.9 shows similar sets of signals in the presence of 0.5 molar equivalents of haemin (yellow) or in the presence of 0.5 molar equivalents of DPIX (blue). Spectra were recorded at 308 K. A similar set of signals is lost to intermediate exchange in each case (unobscured red peaks).

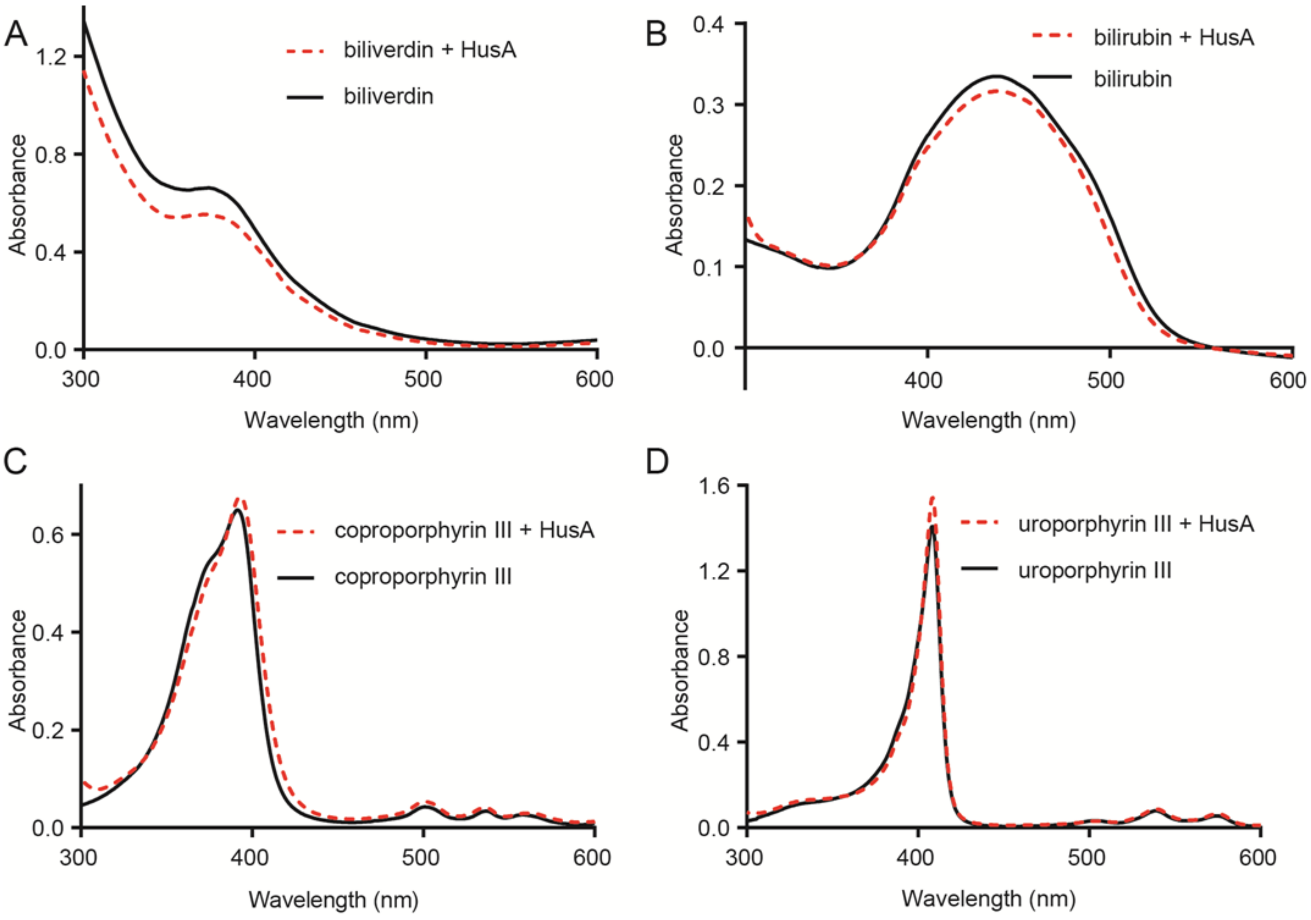

Supplementary Figure 19. UV-vis spectra of 10−20 μM tetrapyrroles, alone and in the presence of 1 to 1.5 molar equivalents of HusA. (**a**) Bilverdin. (**b**) Bilirubin. (**c**) Coproporphyrin III. (**d**) Uroporphyrin III.

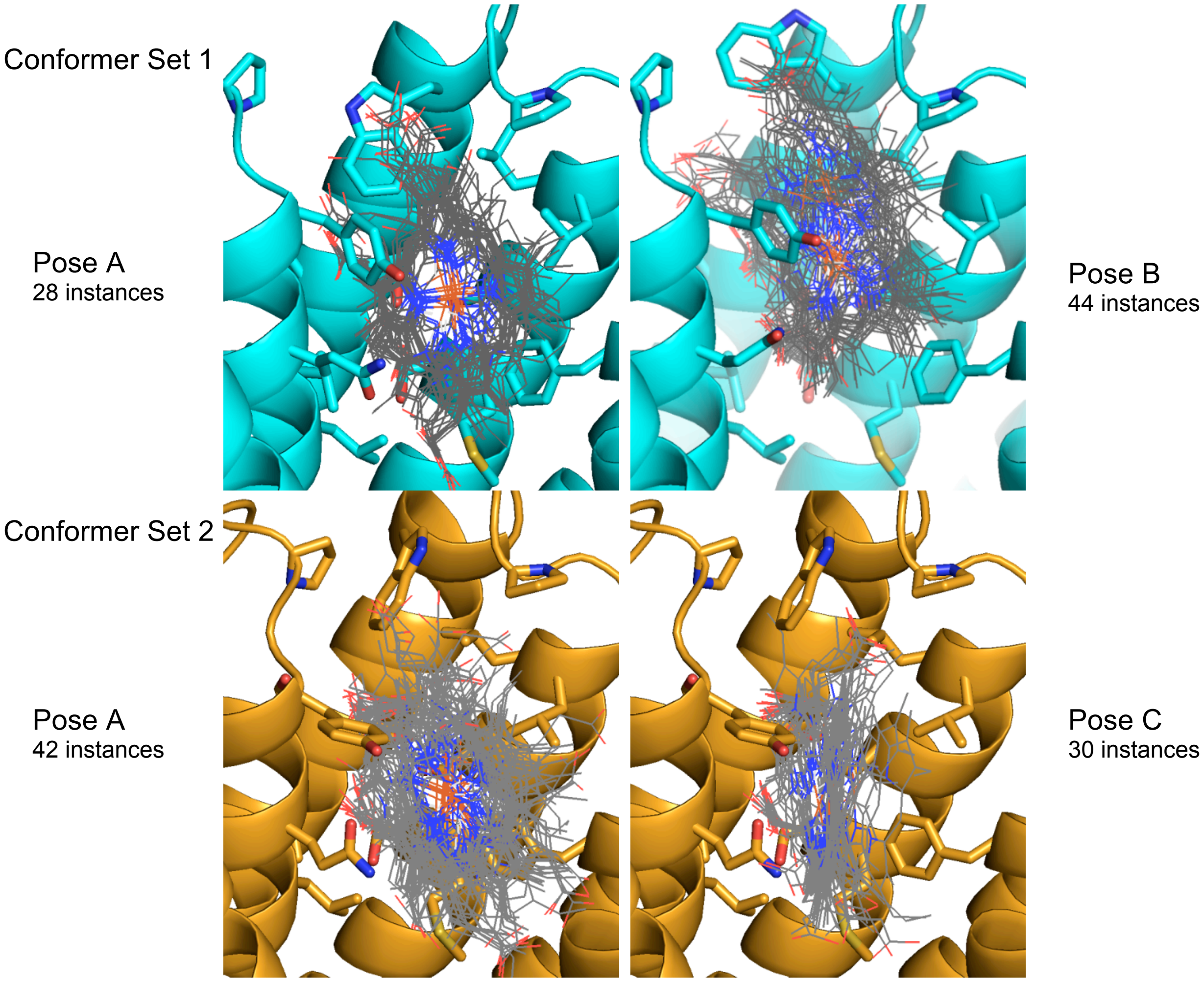

Supplementary Figure 20a. Docking studies of HusA with haemin, PPIX and DPIX. All of the docking solutions for haemin, PPIX or DPIX with apo HusA NMR conformer set 1 (blue) or conformer set 2 (orange) were clustered into one of three poses, Pose A, Pose B or Pose C. Individual docking results (wireframe) are shown for the Poses A, B and C for both HusA NMR conformer sets. Consensus binding poses are shown in Figure 2g. Haemin, PPIX and DPIX docked in the same range of poses, and, hence, the data for these ligands was pooled in this analysis.

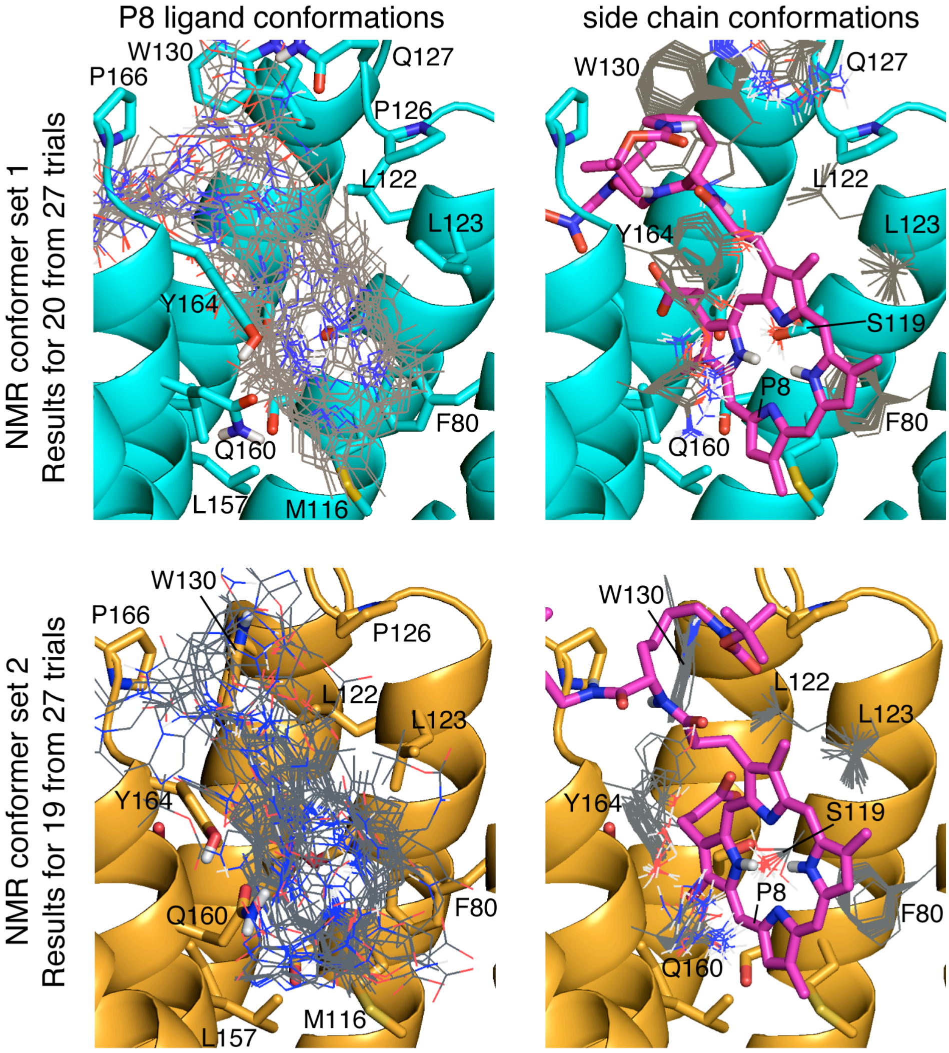

Supplementary Figure 20b. Docking studies of HusA with P8 (DPIX-lysine-metronidazole conjugate). Results are shown for docking P8 to both HusA conformer set 1 (cyan) and conformer set 2 (orange). In total 27 trials were performed against each HusA conformer set. In a total of 20 and 19 dockings the ligand clustered into the poses shown (left panels) for HusA conformer set 1 and conformer set 2, respectively, with the porphyrin moiety of P8 docked at a very similar position to free porphyrins (compare to Figure 2g and Supplementary Figure 20a). A total of 15 docking trials (out of 54) resulted in the lysine-metronidazole group docking into the hydrophobic groove with the porphyrin moiety positioned on the surface of HusA (not shown). The range of side chain rotamers in docking solutions is shown (right panels) with a representative pose of the P8 ligand (sticks) obtained from cluster analysis.

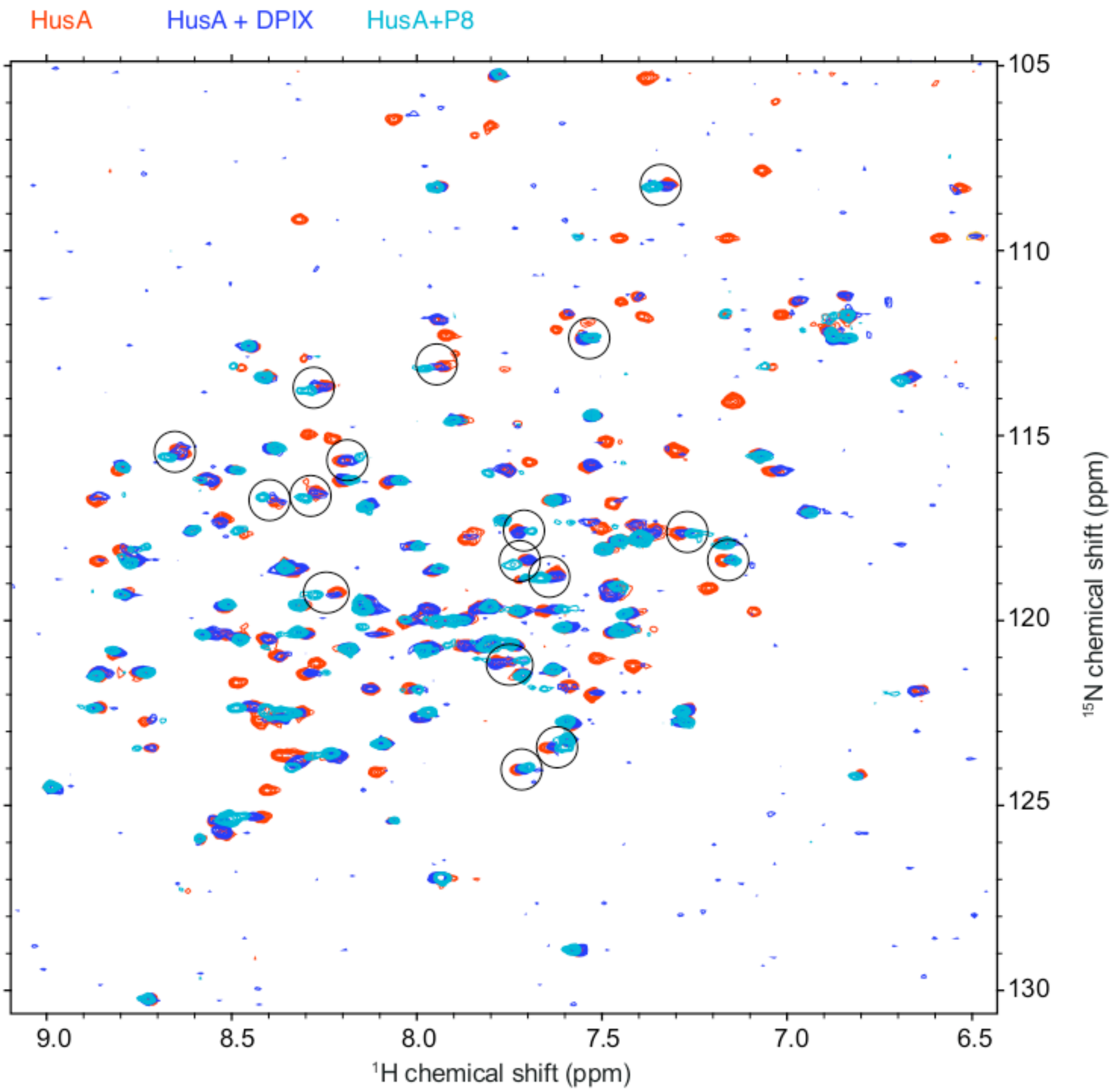

Supplementary Figure 21a. Comparison of spectral changes for HusA upon addition of DPIX or P8. ^{1}H-^{15}N-HSQC spectra of HusA with DPIX or P8. [*U*- ^{13}C, ^{15}N]HusA (160 μM) in 10 mM sodium phosphate, 50 mM NaCl, 5% D_2O, pH 6.9 in the presence of 0.5 molar equivalents of DPIX (red) or 0.5 molar equivalents of P8 (cyan). A similar set of signals is lost to intermediate exchange in each case (unobscured red peaks). For peaks with detectable chemical shift perturbations the shifts show the same trend, but greater magnitude for the P8 titration compared to the DPIX titration. Spectra were recorded at 308 K.

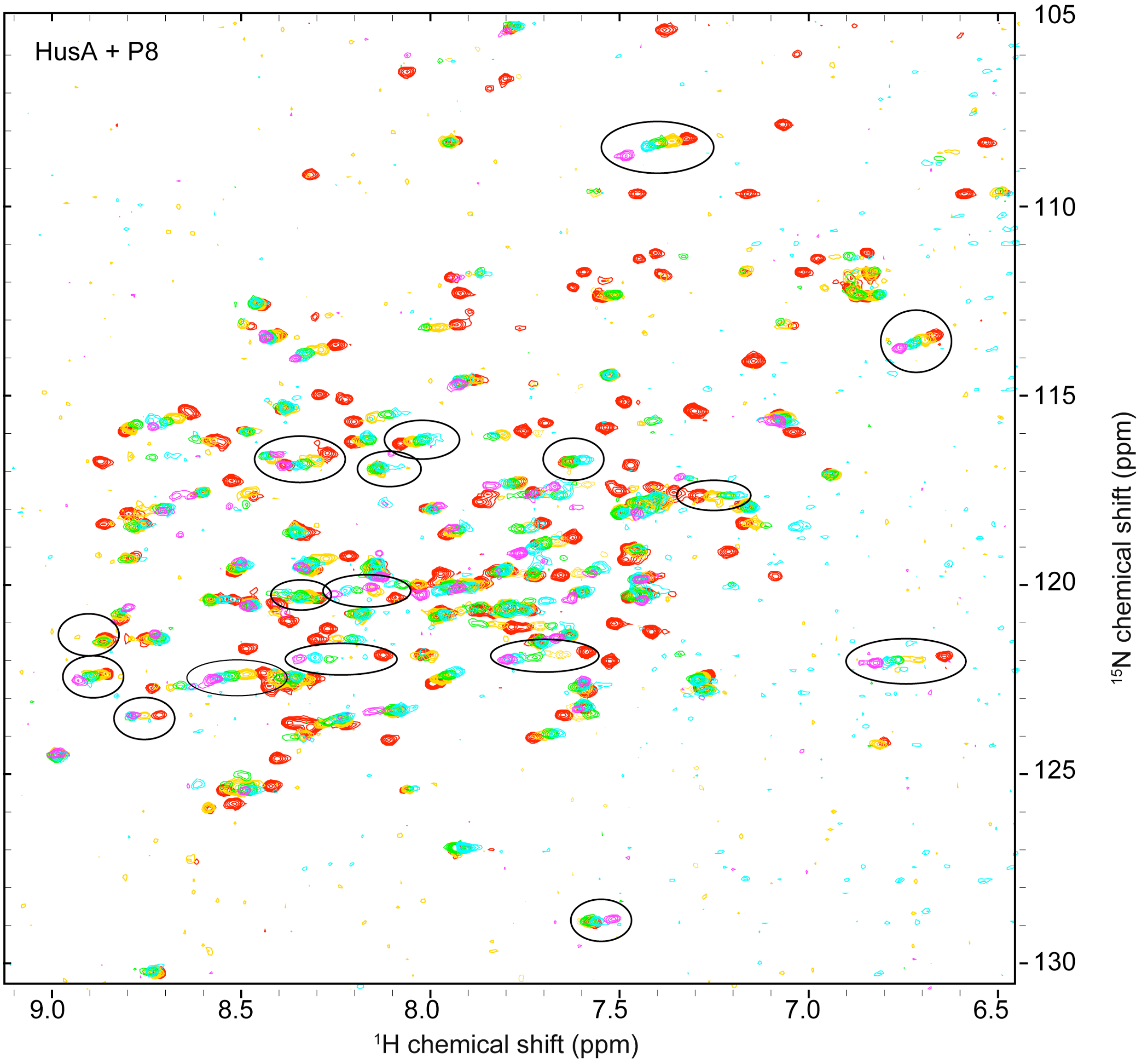

Supplementary Figure 21b. NMR HSQC titration of P8 into HusA, showing peaks with different exchange behaviours. ^{1}H-^{15}N-HSQC spectra are shown for P8 added to HusA at molar ratios: 0 molar equivalents (HusA alone; red), 0.5 molar equivalent (yellow), 1 molar equivalent (green), 2 molar equivalents (cyan), 3 molar equivalents (magenta). Some peaks disappear even at the lowest mixing ratio and do not obviously reappear (red-only peaks), whereas others approximate a fast or fast-intermediate exchange regime (e.g., circled). Comparison with the titration spectrum of HusA + DPIX (Supplementary Figure 11b) shows similar sets of shifts. Spectra recorded at 308 K.

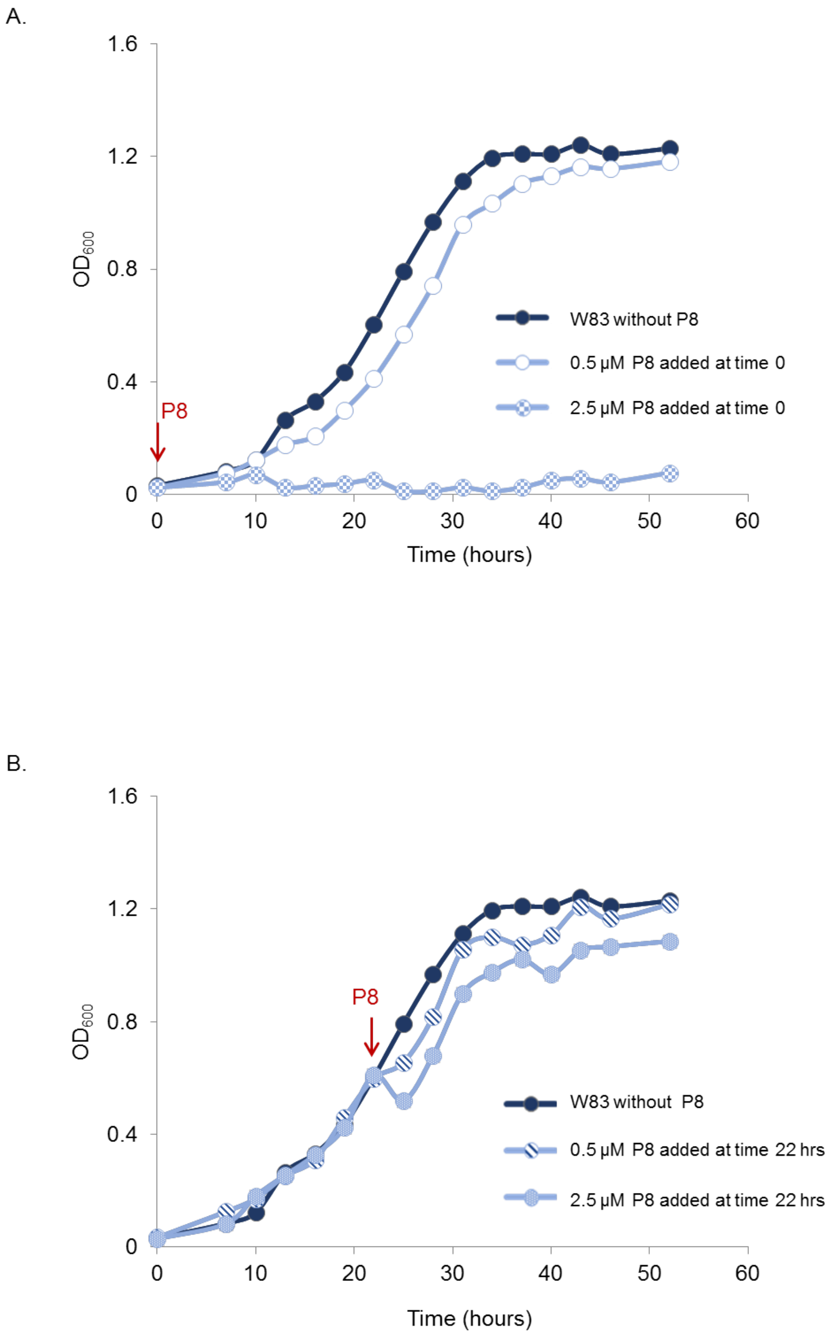

Supplementary Figure 22. Time-kill experiment with high and low densities of *P. gingivalis* wild type W83 in the presence of P8 antibiotic under iron limited condition. (A) Representative planktonic growth curves show control *P. gingivalis* culture without P8 antibiotic (filled dark blue circle), P8 at 0.5 µM (light blue circle) and 2.5 µM (dash-filled light blue circle) added at the commencement of growth. (B) Representative planktonic growth curves shown control *P. gingivalis* culture without P8 antibiotic (filled dark blue circle), P8 at 0.5 µM (dash-filled light blue circle) and 2.5 µM (filled light blue circle) added to the exponentially growing cultures.

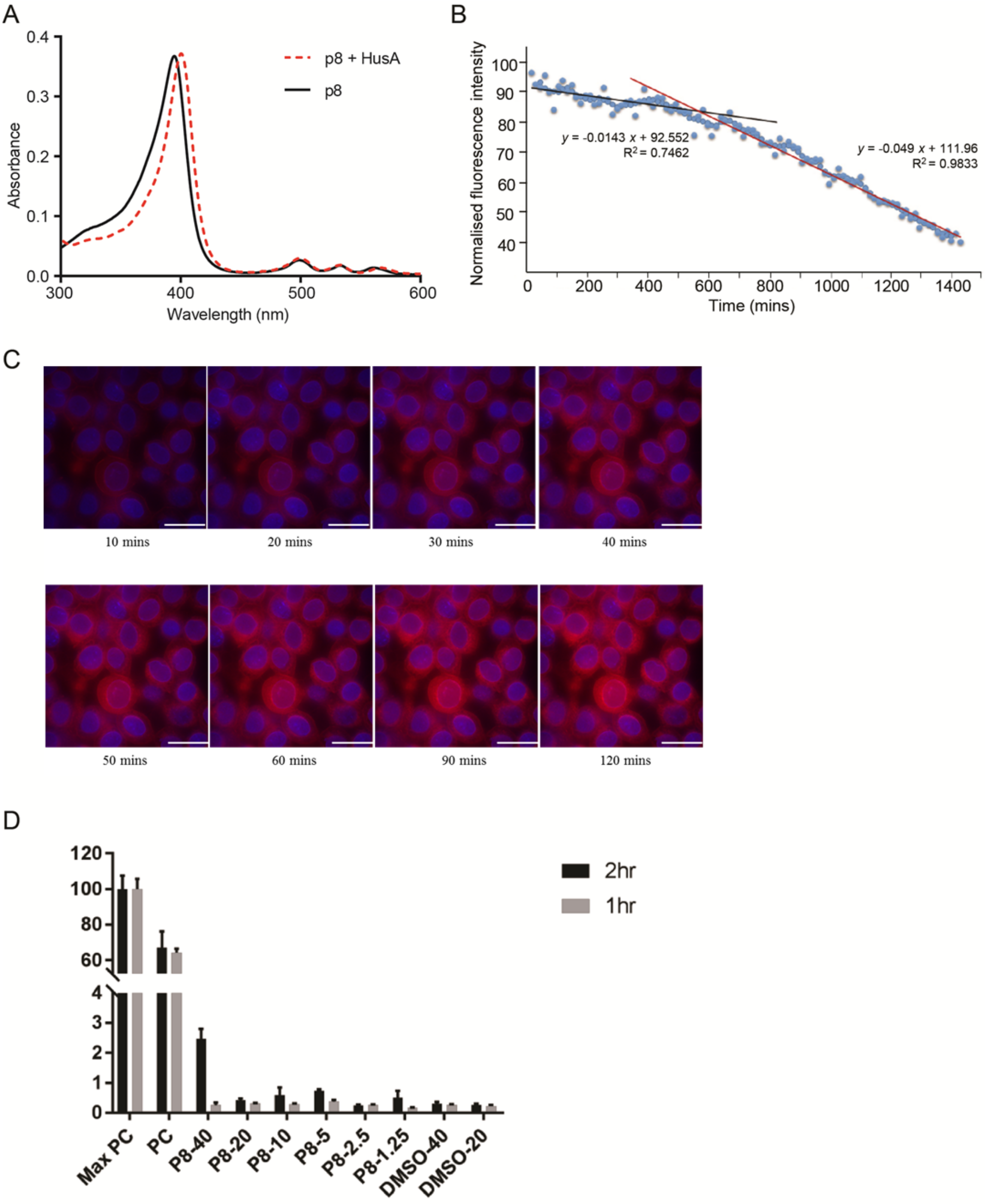

Supplementary Figure 23. (A) UV-vis spectra of 10 μM P8 alone and in the presence of 10 μM HusA. (B) Stability assay of P8 in the presence of gingival epithelial cell lysate. The intensity of P8 fluorescence was applied to monitor the stability of P8 in the presence of cell lysate. (C) Confocal microscope analysis of the progressive uptake of P8 by H413 gingival epithelial cell over a 2 h period. The fluorescent P8 is shown in red and the nuclei stained by DAPI are shown in blue. Scale bar, 25 μm. (D) Lactate dehydrogenase cytotoxicity assay of H413 gingival epithelial cells treated with P8 at various concentrations for 1 h or 2 h incubation. The percentage of the total LDH released into the supernatant was used as an indicator of cytolysis. The results are presented as a percentage of the control in which cells were incubated with PBS buffer only. The assay was conducted in triplicate in two independent experiments.

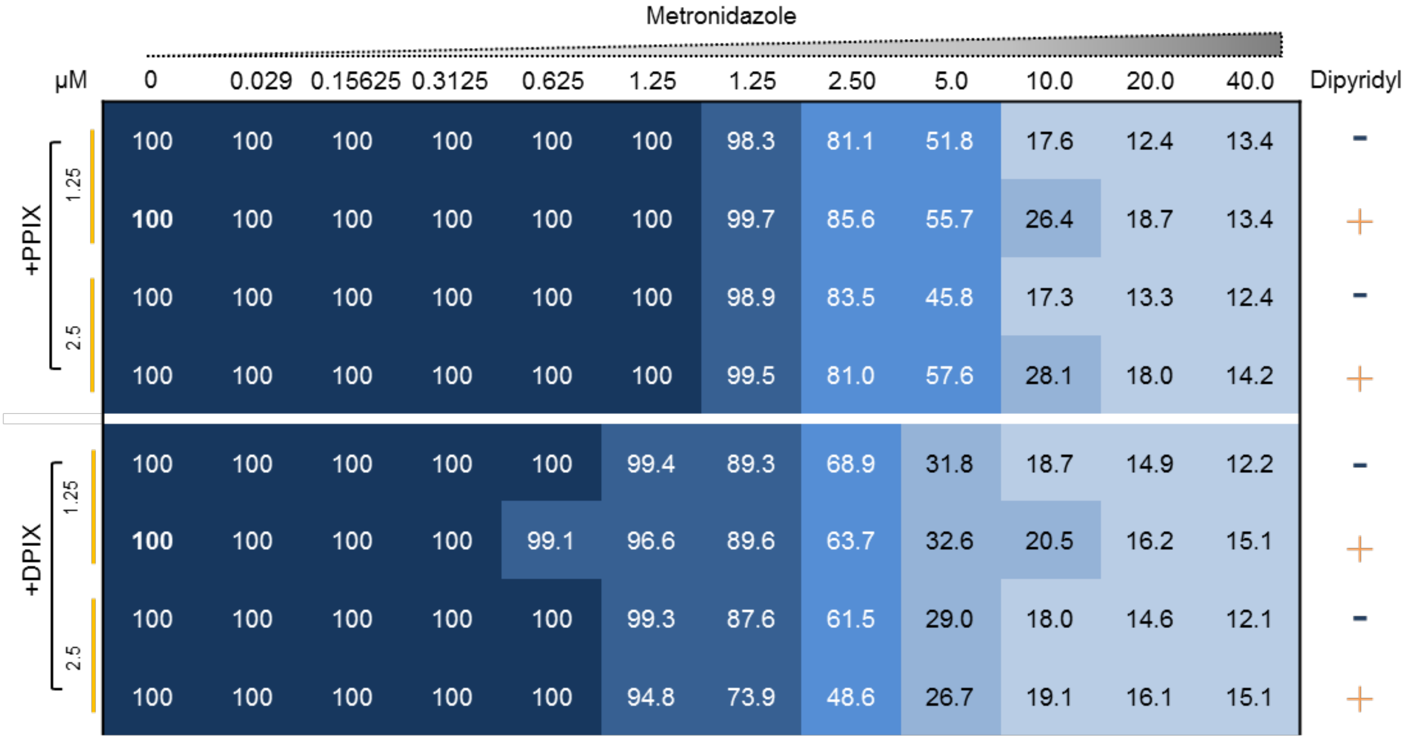

Supplementary Figure 24. Comparison of metronidazole inhibitory effect towards *P. gingivalis* wild-type W83 in the presence of PPIX or DPIX under iron-replete and -limited conditions by checkerboard analysis. Serial dilutions of antibiotics along the horizontal axis were applied to a 96-well plate with *P. gingivalis* W83. Two pre-determined concentrations of PPIX or DPIX were supplemented to evaluate the synergistic effect between metronidazole and porphyrins. Dipyridyl (at 100 μM) was also applied to explore the potential effect under iron-replete and iron-depleted conditions. Absorption at OD_{600} of each well was recorded after 24 h of incubation. The OD_{600} of each strain grown in the absence of antibiotics was defined as 100% growth which was applied to normalise the reading collected from other wells. The normalised percentage of each well was plotted in the checkerboard.

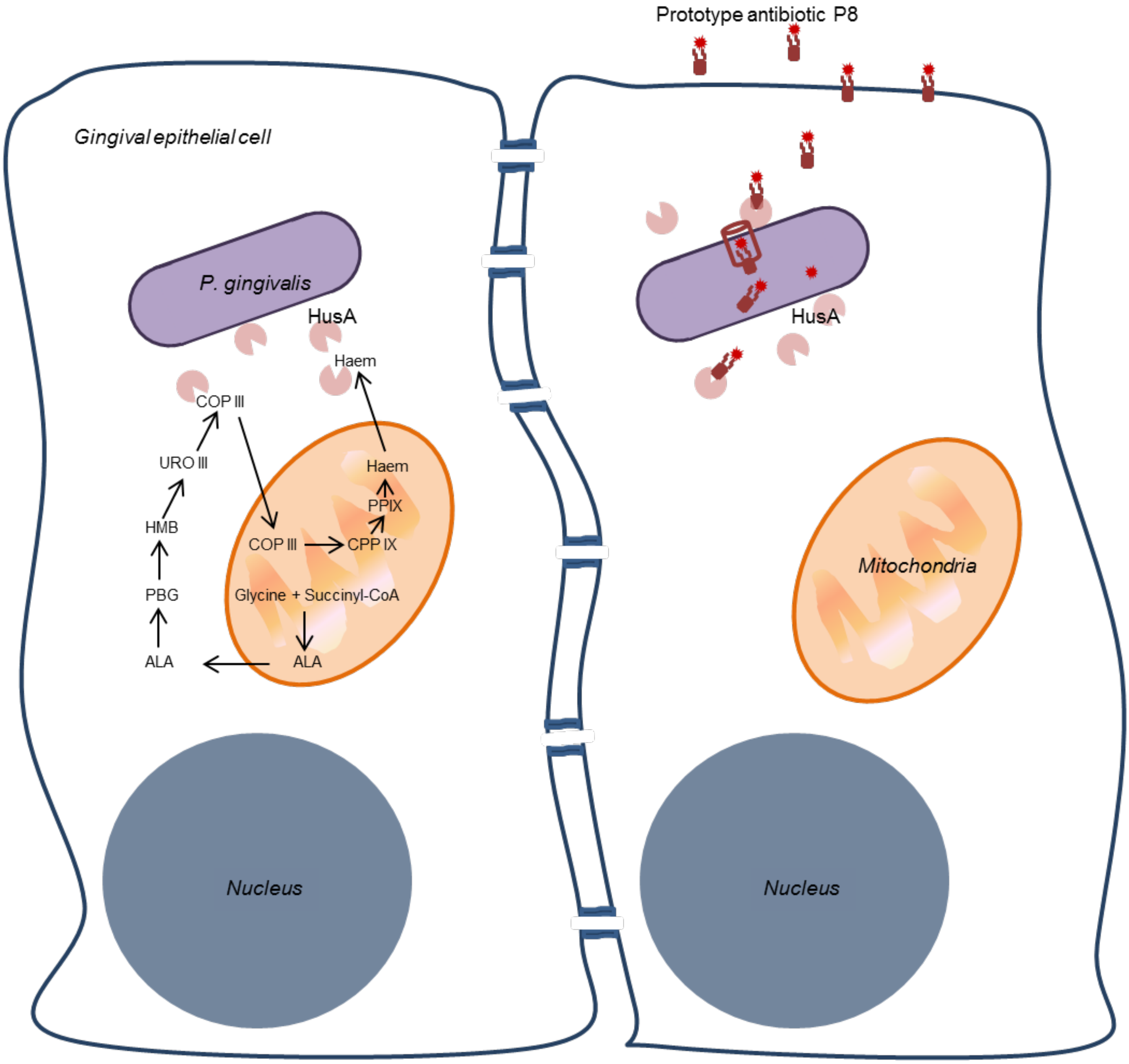

Supplementary Figure 25. Schematic model to illustrate the proposed role of HusA expressed by invading *P. gingivalis* inside gingival epithelial cells and its role in mediating P8 killing. After invading the gingival epithelial cell, *P. gingivalis* is exposed to an intracellular environment in which free iron and porphyrin are restricted. HusA would be expressed at high levels to hijack haem and certain intermediates of the *de novo* haem synthesis pathway. The expressed HusA would then offer an avenue for the deuteroporphyrin IX linked metronidazole (P8) to be captured and uptaken by *P. gingivalis*, thereby achieving a selective killing effect against *P. gingivalis* inside the gingival epithelial cell.

Supplementary Table 1: NMR structure statistics for 20 lowest energy models of HusA

**Completeness of ^{1}H resonance assignments (%)**[a]	Conformer set 1	Conformer set 2
Backbone	99.7	
Side chain	90.2	
Aromatic	98.8	
Stereospecific methyl	76.9	
**Conformationally restricting restraints**[b]		
Distance restraints[c]		
Total	3233	3250
Intraresidue (i = j)	749	743
Sequential (|i – j| = 1)	800	782
Medium range (1 < |i – j| < 5)	827	842
Long range (|i – j| ≥ 5)	857	883
Dihedral angle restraints		
ϕ	121	121
ψ	121	121
Hydrogen bond restraints	0	0
Disulfide restraints	0	0
No. of restraints per residue	17.6	17.7
No. of long range restraints per residue	4.4	4.5
**Residual restraint violations**[b]		
Average no. of distance violations per structure		
0.1–0.2 Å	61.7	58.8
0.2–0.5 Å	19.5	24.3
>0.5 Å	0.3 (0.74 Å max)	1.35 (0.79 max)
Average no. of dihedral angle violations per structure		
1°–10°	0 (0.8° max)	0.6 (2.0° max)
>10°	0	0
**Model quality**[b]		
Rmsd backbone atoms (Å)[d]	0.5	0.5
Rmsd heavy atoms (Å)[d]	0.8	0.9
Rmsd bond lengths (Å)[f]	0.007	0.008
Rmsd bond angles (°)[f]	0.6	0.7
MolProbity Ramachandran statistics		
Most favoured regions (%)	98.4	97.9
Allowed regions (%)	1.1	1.5
Disallowed regions (%)	0.5	0.6
Global quality scores (raw / Z score)		
Verify3D	0.41 / –0.80	0.42 / –0.48
ProsaII	0.90 / 1.03	0.87 / 0.91
Procheck G-factor (ϕ, ψ)[d]	0.56 / 2.52	0.56 / 2.52
Procheck G-factor (all)[d]	0.44 / 2.60	0.44 / 2.60
Molprobity clash score	20.79 / –2.04	21.52 / –2.17
**Model contents**[b]		
Ordered residue ranges[d]	28–60,63–165,168–189,193–199,202–213	28–59,64–165,168–170,172–189,193–213
Total no. of residues	201	201
BMRB accession number	27313	27313
PDB ID code	6BQS	6CRL

[a] Computed using AVS software [2]
[b]Analysed using the Protein Structure Validation Suite (PSVS)[3] over 197 conformationally restricted residues
[c] Calculated using sum over r^{-6}
[d] Residues with the sum of dihedral angle order parameters $s(\phi) + s(\psi) > 1.8$
[f]relative to standard dictionary

Supplementary Table 2: SAXS Results for HusA and HusA:haem

**SAXS data collection parameters**			
Source, instrument and reference for data acquisition and processing details	Australian synchrotron SAXS-WAXS Beamline, http://www.synchrotron.org.au/aussyncbeamlines/saxswaxs		
Wavelength	1.0332 Å		
Beam geometry	250 x 130 μm, 2.683 m camera length		
q-measurement range ($Å^{-1}$ or nm^{-1})	0.00663–0.3104 $Å^{-1}$		
Absolute scaling method	Comparison with scattering from pure H_2O		
Normalization of SAXS data	Each measurement frame is normalized to transmitted intensity measured by beam stop counter followed by scaling to the scattering of water (for absolute scaling) [4]		
Monitoring for radiation damage	Frame by frame comparison as judged visually (note the applied dose to each sample is <~210 Gy so radiation damage is relatively rare)		
Exposure time	1s × 24		
Sample configuration	Static		
Sample temperature (°C)	22 °C		
**Sample details**			
Sample description: sequence (including non-native residues after thrombin cleavage in red, and extra residue from cloning in blue)<br>UniProt sequence ID<br>organism	GQGTAYAEVMNRKVAALDSVPPTEYATLAADFSRIAAVEGSDWM AAYYTAYCRIIPAFGNPSEADRLCEEAESMLSKAESLGGDLSEIACL RSMAASARLLVNPQERWQTYGAESSRQLAVALEANPANPRAYFLQ AQSLLYTPAQFGGGKDKALPFAEKSVSCYAAATVSPAYAPHWGEQ QARQLLMLCKAETQELVPR;<br>UniProt: Q7MSY3, residues 24-218<br>*Porphyromonas gingivalis* gene PG_2227<br><br>Non-native residues from cloning (at N-terminus) and thrombin cleavage (at C-terminus) are coloured in blue and red, respectively.<br><br>Samples containing haem had 1:1 molar equivalents of haemin added; $C_{34}H_{33}N_4O_5Fe$		
Source	Bacterially expressed (see Methods) protein;<br>haemin from hematin purchased from Sigma H3505		
Organism, UniProt sequence ID (residues in construct)	*Porphyromonas gingivalis*, product from *PG2227*		
Extinction coefficient (A280, 0.1% w/v solution)	1.454		
Partial specific volume from sequence (cm^3 g^{-1})	0.733		
Particle contrast from sequence and solvent constituents (ρ_{sample}-,$\rho_{solvent}$; 10^{10} cm^{-2})	2.885 apo HusA (reduced buffer)<br>2.884 apo HusA (standard buffer)<br>2.822 HusA:heam (standard buffer)		
Molecular mass from sequence (Da)	21596.4 (protein monomer)<br>22229.9 (for a 1:1 molar ratio monomeric protein:haem<br>(haem is 633.5 Da)		
Solvent blank	50 mM TRIS, 150 mM NaCl, 2 mM TCEP, pH 8, dialysate after dialysis.		
**SAXS Data analysis**			
	HusA<br>(reduced buffer)	HusA<br>(standard buffer)	HusA:haem<br>(standard buffer)
Sample concentrations measured (mg/mL)	0.8, 1.6, 3.2, 6.4	1.5, 3, 5.9	3

**Guinier analysis***			
$I(0)$ (cm^{-1}) (as measured in mg/mL)[a]	0.015 ± 0.00003<br>0.030 ± 0.00003<br>0.060 ± 0.00004<br>0.120 ± 0.00006<br>Averaged data<br>0.015 ± 0.00001	0.027 ± 0.00007<br>0.057 ± 0.00001<br>0.120 ± 0.0002	0.061 ± 0.00004
R_g (Å)	20.08 ± 0.09<br>19.91 ± 0.04<br>20.18 ± 0.03<br>20.42 ± 0.02<br>Merged data<br>20.05 ± 0.03	21.89 ± 0.09<br>21.76 ± 1.16<br>22.75 ± 0.08	23.01 ± 0.29
qR_g max	1.2 for all concentrations	1.2, 1.13, 1.05 for 1.5, 2.95, 6.92 mg/mL data	1.2
$q_{\min}$ ($Å^{-1}$)	0.011 for averaged data	0.025	0.018
Quality of fit (Pearson's R)	averaged data<br>0.999	1.5 mg/mL data<br>0.994	0.989
Molecular Weight from $I(0)$ (ratio to expected value from sequence)[b]	averaged data<br>25248 (1.17)	1.5 mg/mL data<br>24256 (1.12)	28617(1.29)
*Results are presented in ascending order of concentration from top to bottom of the column for each parameter, with the result for the merged data set listed last for the samples in reduced buffer.			
P*(*r*) analysis*			
$I(0)$ (cm^{-1}) (normalised to unit C in mg/mL)	0.01845	0.01804	0.01985
R_g (Å)	20.51 ± 0.03	21.73 ± 0.09	22.49 ± 0.17
d_{max} (Å)	80	80	
q range ($Å^{-1}$)	0.0138–0.2398	0.0248–0.2398	0.0333–0.2398
χ^2 (total estimate from GNOM)	3.15 (0.71)	1.54 (0.76)	0.40 (0.70)
Molecular Weight from $I(0)$ (ratio to expected value from sequence)	24845 (1.15)	24310 (1.13)	27937(1.26)
Porod Volume ($Å^{-3}$), M_r estimate from $(2/3)V_p$	30700,<br>20466	31100,<br>20730	34500,<br>23000
**Results are for the averaged data set for the apo HusA samples in reduced buffer, 1.5 mg/mL for the apo HusA sample in standard buffer, 3 mg/mL for the HusA sample with haem in standard buffer			
**Model fitting results**			
**Bead Modelling – DAMMIF**			
q range for fitting ($Å^{-1}$)	0.0138–0.2398	0.0248–0.2398	0.0333–0.2398
Resolution (Å) (from DAMRES)	23 ± 2	23 ± 2	22 ± 2
Symmetry, anisotropy assumptions	P1, none	P1, none	P1, none
Ambiguity measure - Normalised Spatial Discrepancy, NSD (standard deviation) between models, number of clusters for 20 calculations for NSD > 0.7	0.806(0.101)	0.785 (0.102)	0.700(0.97)
χ^2 range for 20 calculations, constant adjustment to intensities	3.21-3.28, 0.00002	1.54-1.55, 0.0001	0.41, 0.0002
M estimate for shape models (Da)	20600	20900	23300
**Bead Modelling – DAMMIN**			

q range for fitting (Å^{-1})	0.0138–0.2398	0.0248–0.2398	0.0333–0.2398
Symmetry, anisotropy assumptions	P1, none	P1, none	P1, none
χ^2, CorMap P value<br>constant adjustment to intensities	3.15, 0.02, 0.0004	1.55, 0.12, 0.0001	0.40, 0.02, 0.0002

**Software employed for SAXS data reduction, analysis and interpretation**	
SAXS data reduction to sample minus solvent, including desmearing, extrapolation, merging, etc as relevant	ScatterBrain 8.22[b] (http://www.synchrotron.org.au/aussyncbeamlines/saxswaxs/software-saxswaxs) for data reduction to $I(q)$ *vs q* and PRIMUS from ATSAS 2.8.0 [5] for solvent subtraction
Intensity scaling method	Absolute scaling based on H_2O scattering
Extinction coefficient estimate	ProtParam [6]
Calculation of *Äñ* and values from sequence	MULCh 1.1 (06/10/16) [7]
Basic analyses: Guinier, $P(r)$, Porod volume	PRIMUSQT from ATSAS 2.8.0 program suite [8]
Shape/bead modelling	DAMMIF [9] and DAMMIN [10] via ATSAS on-line https://www.embl-hamburg.de/biosaxs/atsas-online/
Model profile from HusA solution structure	CRYSOL [11]
3D graphic model representations	PyMOL v1.70.0.5 Win64

[a] Errors in $I(0)$ are propagated counting statistics only. Errors smaller than the number of decimal places for the values are a consequence of the format of the output from ASAS 2.8.0.

[b] This version of ScatterBrain outputs 2× standard errors. The errors reported here are as obtained from ScatterBrain and were used directly in all subsequent analyses.

Errors in $I(0)$ are propagated counting statistics only. Concentration values are based on A280 measurements using a Nanodrop and the calculated extinction coefficients with a combined estimated error of ~10%.

Supplementary Table 3: Binding affinities of HusA and mutant proteins to haemin as determined using tryptophan fluorescence quenching assays

**Protein**	**K_d (μM)**
HusA wild-type	7.3 ± 1.1
L107A	5.5 ± 0.3
R121A	6.7 ± 1.2
L123N-V124N	10.3 ± 0.7
W130N	16.0 ± 1.8
Y133A	7.4 ± 0.6
R139A	6.3 ± 1.4
Y164A	13 ± 0.7
NNNA	N.D.

Supplementary Table 4: Binding affinities of HusA to haemin and porphyrin analogues

**Ligand**	**K_d (μM)**
Haemin	7.3 ± 1.1
PPIX	1.9 ± 0.6
Zn(II)-PPIX	1.3 ± 0.4
Fe(III)-DPIX	0.22 ± 0.05
DPIX	0.36 ± 0.09

Supplementary Table 5: Change in $^{1}H,^{15}N$-HSQC peak heights at HusA:porphyrin ratio 2:1

	Change in peak height ln(bound/free)				
	Buffer 298 K	Zn-PPIX 298 K	Haemin 298 K	DPIX 308 K ‡	P8 308 K §
THR26	NA	NA	0.06	NA	NA
ALA27	NA	NA	0.00	NA	NA
TYR28	NA	NA	-0.04	NA	NA
ALA29	0.17	0.00	-0.15	-0.18	-0.25
GLU30	0.11	0.00	-0.23	0.00	-0.20
VAL31	0.10	0.00	-0.04	-0.11	-0.44
MET32	NA	NA	NA	-0.17	-0.41
ASN33	0.29	0.25	-0.16	-0.29	-0.14
ARG34	0.08	-0.09	-0.21	0.16	-0.11
LYS35	0.26	0.06	-0.34	-0.07	-0.17
VAL36	0.00	0.00	-0.24	0.22	-0.41
ALA37	0.11	0.08	-0.30	-0.27	-0.30
ALA38	0.18	0.09	-0.49	-0.19	-0.66
LEU39	0.16	-0.09	-1.04	-0.22	-1.43
ASP40	NA	NA	NA	NA	-1.35
SER41	0.43	0.19	-0.65	-0.25	-0.97
VAL42	0.23	-0.06	-0.92	-0.26	-2.17
THR45	0.98	0.41	-1.00	NA	NA
GLU46	0.29	0.13	-0.32	-0.24	-0.44
TYR47	0.27	0.08	0.26	-0.31	-0.29
ALA48	1.10	0.22	0.14	NA	NA
THR49	0.66	0.25	0.03	NA	NA
LEU50	0.29	0.00	-0.03	0.00	-0.50
ALA51	NA	NA	NA	NA	-1.01
ALA52	NA	NA	NA	-0.18	-1.21
ASP53	0.19	0.12	0.02	0.13	-0.23
PHE54	0.32	0.00	0.03	0.00	-0.47
SER55	0.31	0.04	-0.03	-0.07	-0.27
ARG56	0.28	0.09	-0.15	-0.21	-0.39
ILE57	0.16	-0.13	-0.15	-0.33	-1.08
ALA58	0.08	-0.04	-0.13	0.11	-0.24
ALA59	0.22	0.03	-0.11	-0.18	-0.28
VAL60	0.00	-0.06	-0.04	-0.22	-0.20
GLU61	0.74	0.31	-0.06	-0.81	-0.18
GLY62	1.43	0.69	0.00	NA	NA
SER63	0.42	0.17	0.03	-0.12	-0.28
ASP64	0.27	0.00	0.00	-0.13	-0.21
TRP65	0.14	-0.08	0.13	-0.32	-0.31
MET66	0.20	0.05	0.06	0.00	-0.10
ALA67	0.00	0.00	0.05	0.06	-0.22
ALA68	-0.04	-0.04	0.02	0.00	-0.34
TYR69	0.06	-0.22	0.04	0.00	-0.59
TYR70	0.19	-0.07	0.04	-0.20	-0.24
THR71	-0.19	-0.35	-0.04	-0.56	-0.69
ALA72	0.57	0.48	-0.12	0.22	-1.34
TYR73	NA	NA	NA	-0.45	-2.30
CYS74	0.13	-0.14	-0.31	-0.09	-1.45
ARG75	0.11	0.00	-0.32	-0.56	-1.85
ILE76	0.09	-0.79	-1.56	-1.39	-1.54
ILE77	0.19	-0.24	-1.23	-1.10	-4.00
ALA79	0.00	-0.93	-1.44	-2.20	-2.83
PHE80	0.03	-2.05	-1.34	-2.05	-3.89
GLY81	0.44	-0.59	-1.67	-1.79	-4.00
ASN82	0.15	-0.33	-1.10	-0.79	-4.00
SER84	0.69	0.06	-0.98	NA	-0.81
GLU85	0.29	-0.33	-0.92	-0.63	-1.10
ALA86	0.15	-0.92	-1.17	-0.75	-4.00
ASP87	0.24	-0.88	-1.69	-0.98	-1.91
ARG88	0.23	-0.41	-0.89	-1.05	-1.64
LEU89	0.29	-0.22	-0.97	-0.61	-1.65
CYS90	NA	NA	NA	-1.10	-0.97
GLU91	NA	NA	NA	-2.50	-3.50
GLU92	NA	NA	NA	-0.11	-2.80
ALA93	NA	NA	NA	-0.41	-1.76
GLU94	0.39	-0.89	-1.13	-1.18	-1.39
SER95	NA	NA	NA	NA	-2.05
MET96	0.45	-0.56	-0.88	-0.69	-0.94

LEU97	0.29	-0.29	-0.48	-0.51	-1.39
SER98	0.14	-0.17	-0.31	-0.58	-0.92
LYS99	0.12	-0.36	-0.78	-0.31	-0.48
ALA100	0.26	-0.18	-0.41	-0.14	-0.41
GLU101	0.15	-0.18	-0.13	0.00	-0.18
SER102	NA	NA	NA	NA	-0.74
LEU103	0.16	0.34	-0.03	-0.08	-0.34
GLY104	0.29	0.15	0.03	-0.17	-0.24
GLY105	0.43	0.07	-0.08	0.10	-0.35
LEU107	0.23	-0.05	-0.04	-0.07	-0.29
SER108	0.09	-0.15	-0.29	0.08	-0.34
GLU109	0.08	-0.29	-0.21	-0.98	-0.58
ILE110	0.24	-0.10	-0.38	-0.51	-0.88
ALA111	0.13	-0.20	-0.13	-0.17	-0.31
CYS112	0.16	-0.19	-0.41	-0.34	-0.48
LEU113	0.12	-0.21	-0.37	-0.56	0.21
ARG114	0.13	-0.41	-0.31	0.00	-0.31
SER115	NA	NA	NA	-1.32	-1.06
MET116	NA	NA	NA	-0.51	-1.25
ALA117	NA	NA	NA	NA	-1.01
ALA118	0.17	-0.30	-1.02	-0.59	-1.35
SER119	NA	NA	NA	-0.31	-1.20
ALA120	0.27	-1.67	-1.39	-2.48	NA
ARG121	NA	NA	NA	-1.39	-2.56
LEU122	NA	NA	NA	NA	-2.94
LEU123	0.11	-1.87	-1.56	-2.50	-3.09
VAL124	0.00	-1.50	-2.05	-2.50	-4.00
ASN125	0.11	-3.26	-1.73	-2.50	-4.00
GLN127	0.27	-1.67	-1.99	-2.08	-2.35
GLU128	0.03	-2.05	-1.34	-2.05	-3.89
ARG129	0.17	-1.67	-1.10	-2.50	-2.94
TRP130	0.12	-1.75	-1.50	-2.50	-4.00
THR132	0.48	-1.53	-1.33	-2.20	-4.00
TYR133	0.25	-2.20	-1.30	-2.50	-3.18
GLY134	0.25	-1.50	-1.75	-2.50	-1.85
ALA135	0.33	-0.36	-1.10	-0.47	-0.49
GLU136	0.13	-0.79	-0.37	-0.69	-0.39
SER137	0.17	-0.74	-0.64	-0.59	-1.32
SER138	0.26	-1.91	-1.59	-2.08	-3.40
ARG139	0.26	-0.69	-0.72	-0.58	-4.00
GLN140	NA	NA	NA	NA	-0.88
LEU141	NA	NA	NA	-1.39	-1.21
ALA142	0.26	-1.10	-1.12	-0.18	-1.85
VAL143	0.20	-0.04	-0.39	-1.20	-0.57
ALA144	0.12	-0.83	NA	-0.31	-1.01
LEU145	0.00	-1.20	-0.79	-1.22	-1.10
GLU146	0.19	-0.11	-0.99	-0.69	-0.89
ALA147	NA	NA	NA	-0.36	-0.23
ASN148	0.20	-0.43	-0.69	0.00	-0.76
ALA150	0.20	-0.04	-0.24	0.00	-0.62
ASN151	0.15	0.05	-0.21	-0.07	-0.58
ARG153	NA	NA	NA	-0.61	-0.96
ALA154	0.22	-0.36	-1.01	-0.56	-1.75
TYR155	0.17	-0.13	-0.79	0.00	-0.79
PHE156	NA	NA	NA	-0.18	-1.39
LEU157	NA	NA	NA	NA	-0.74
GLN158	0.00	-1.10	-1.55	-0.69	-2.20
ALA159	NA	NA	NA	-2.50	-3.33
GLN160	0.21	-0.53	-1.87	-0.44	-4.00
SER161	-0.04	-1.75	-1.58	-2.35	-2.67
LEU162	0.22	-1.39	-1.87	-0.85	-1.83
LEU163	NA	NA	NA	-2.14	-2.60
TYR164	0.11	-1.87	-1.56	-2.50	-3.09
THR165	0.04	-2.48	-1.48	-1.87	-3.50
PHE169	NA	NA	NA	-1.67	-1.48
GLY170	0.41	-0.59	-0.53	-2.08	-2.83
GLY171	0.25	-1.66	-1.32	-2.48	-3.09
GLY172	0.14	-1.39	-1.98	-2.50	-4.00
LYS173	1.45	-0.29	-2.56	NA	NA
ASP174	0.31	-0.46	-1.22	-0.24	-0.65
LYS175	0.17	-0.79	-0.97	-0.63	-1.02
ALA176	NA	NA	NA	-0.86	-1.46
LEU177	0.30	-0.75	-1.06	-2.20	-3.18
PHE179	0.09	-0.15	-0.21	-0.13	-0.41
ALA180	0.23	-0.31	-0.37	-0.34	-0.65

GLU181	0.07	-0.69	-0.59	-0.64	-1.54
LYS182	0.17	-0.10	0.00	0.00	-0.35
SER183	0.00	-0.24	-0.16	-0.41	-0.26
VAL184	0.00	-0.13	-0.26	0.00	-0.38
SER185	0.21	-0.55	-0.69	-0.97	-0.89
CYS186	NA	NA	NA	0.00	-0.30
TYR187	0.24	-0.07	-0.62	-0.36	-0.51
ALA188	0.11	-0.22	-0.10	-0.51	-0.44
ALA189	0.20	-0.20	0.02	-0.15	-0.29
ALA190	0.16	-0.03	-0.32	-0.33	-0.44
THR191	0.79	0.00	-0.08	NA	-0.32
VAL192	0.81	0.06	-0.39	NA	NA
SER193	NA	NA	NA	-0.47	-0.44
ALA195	NA	NA	NA	0.00	-0.31
TYR196	0.05	0.00	-0.34	-0.08	-0.57
ALA197	0.30	0.10	-0.31	0.00	-0.30
HIS199	NA	NA	NA	NA	-0.98
TRP200	0.10	-0.36	-0.47	-0.85	-4.00
GLY201	0.62	0.13	-0.61	NA	NA
GLU202	NA	NA	NA	-0.48	-1.21
GLN203	NA	NA	NA	-0.37	-1.39
GLN204	0.26	0.05	-0.30	-0.22	-0.10
ALA205	0.26	0.06	-0.27	0.06	-0.74
ARG206	NA	NA	NA	NA	-0.74
GLN207	NA	NA	NA	-0.11	-3.53
LEU208	NA	NA	NA	-0.88	-0.80
LEU209	NA	NA	-0.76	-0.37	-1.65
MET210	0.07	-0.21	-1.15	-0.22	-2.08
LEU211	0.16	-0.24	-0.89	-0.33	-1.08
CYS212	0.12	-0.23	-1.19	-0.26	-1.39
LYS213	0.17	-0.20	-0.93	-0.22	-1.15
ALA214	0.39	-0.10	-1.41	-0.51	-0.83
GLU215	NA	NA	NA	NA	-0.69
THR216	1.45	0.34	-1.41	NA	NA
GLU218	NA	NA	NA	-1.22	-1.30
VAL220	0.39	-0.15	-1.46	-0.35	-0.72
ARG222	0.34	0.29	-1.30	-0.13	-0.31

NA: no data due to signal overlap or signal absence in free HusA

‡ Log_e peak height ratio truncated at –2.5, equivalent noise level in the bound spectrum.

§ Log_e peak height ratio truncated at –4.0, equivalent noise level in the bound spectrum.

Supplementary Table 6: Primers used to construct recombinant HusA mutants

Mutant	Primer name	Sequence (5' - 3')	Reference
L107N	HusAL107NFA	GGCGGAGATAACTCCGAAATAGCCTGTTTGCG	This study
	HusAL107AFB	ATAGCCTGTTTGCGTAGCAT	This study
	HusAL107NRA	TTCGGAGTTATCTCCGCCGAGGGATTC	This study
	HusAL107ARB	GAGGGATTCCGCTTTGCT	This study
R121A	HusAR121AFA	GCAGCCCTTTTGGTCAATCCGCAAGAACGCTGGCAG	This study
	HusAR121AFB	CCGCAAGAACGCTGGCAG	This study
	HusAR121ARA	ATTGACCAAAAGGGCTGCCGAGGCGGCCATGCTACG	This study
	HusAR121ARB	CGAGGCGGCCATGCTACG	This study
LV123/4NN	HusALV123NNFA	GCACGCCTTAATAACAATCCGCAAGAACGCTGGCAG	This study
	HusAR121AFB	CCGCAAGAACGCTGGCAG	This study
	HusALV123NNRA	ATTGTTATTAAGGCGTGCCGAGGCGGCCATGCTACG	This study
	HusAR121ARB	CGAGGCGGCCATGCTACG	This study
W130N	HusAW130NFA	GAACGCAACCAGACATATGGAGCAGAGAGCAGCCGA	This study
	HusAW130NFB	GGAGCAGAGAGCAGCCGA	This study
	HusAW130NRA	ATATGTCTGGTTGCGTTCTTGCGGATTGACCAAAAGGC	This study
	HusAW130NRB	TTGCGGATTGACCAAAAGGC	This study
Y133A	HusAY133AFA	GAACGCTGGCAGACAGCTGGAGCAGAGAGCAGCCGA	This study
	HusAW130NFB	GGAGCAGAGAGCAGCCGA	This study
	HusAY133ARA	AGCTGTCTGCCAGCGTTCTTGCGGATTGACCAAAAGGC	This study
	HusAW130NRB	TTGCGGATTGACCAAAAGGC	This study
R139A	HusAR139AFA	AGCAGCGCACAGTTGGCTGTCGCCCTTGAAGCCAAC	This study
	HusAR139AFB	GTCGCCCTTGAAGCCAAC	This study
	HusAR139ARA	AGCCAACTGTGCGCTGCTCTCTGCTCCATATGTCTGCCAG	This study
	HusAR139ARB	CTCTGCTCCATATGTCTGCCAG	This study
Y164A	HusAY164AFA	CTGCTGGCTACCCCTGCGCAGTTCGGAGGGGGCAAG	This study
	HusAY164AFB	CAGTTCGGAGGGGGCAAG	This study
	HusAY164ARA	CGCAGGGGTAGCCAGCAGGCTTTGGGCCTGCAGGAA	This study
	HusAY164ARB	GCTTTGGGCCTGCAGGAA	This study
Y164F	HusAY164FFA	CTGCTGTTTACCCCTGCGCAGTTCGGAGGGGGCAAG	This study
	HusAY164FFB	CAGTTCGGAGGGGGCAAG	This study
	HusAY164FRA	CGCAGGGGTAAACAGCAGGCTTTGGGCCTGCAGGAA	This study
	HusAY164FRB	GCTTTGGGCCTGCAGGAA	This study
NNNA	HusALV123NW130NFA	AATAACAATCCGCAAGAACGCAACCAGACATATGGAGCAGAGAGCAGCCG	This study

	HusALV123AW130N FB	CAGACATATGGAGCAGAGAGCAGCCGACA	This study
	HusALV123NW130N RA	GTTGCGTTCTTGCGGATTGTTATTAAGGCGTGCCGAGGCGGC	This study
	HusALV123AW130N RB	AAGGCGTGCCGAGGCGGC	This study
Sequencing primers	T7pmtF	CGAAATTAATACGACTCACTATAGG	Gao et al [12]
	T7terR	TATGCTAGTTATTGCTCAGCG	Gao et al [12]

Supplementary Table 7: Primers and probes used for real time PCR

Genes	Primer /probe name	Sequence (5' - 3')	Reference
husA	FAMRT2227P	TCCCGGCCTTCGGCAACCCCTC	Gao et al [12]
	RT2227F	CACACTGGCTGCGGACTT	Gao et al [12]
	RT2227R	CCGCTTTGCTCAGCATGGAT	Gao et al [12]
hmuY	FAMRThmuYP	TCACGGTCCTGCCGGTCCCACTTACAAGCTGAGC	This study
	RThmuYF	GGCAAGAAGAACGCACAGGGATT	This study
	RThmuYR	CATCAGCACCACGAACGAAGAAGAC	This study
hmuR	FAMRThmuRP	CCTACGCGGAAGGGTATCGTGCCCCCTC	This study
	RThmuRF	TGCCATGTACAAGTGCAGCCAT	This study
	RThmuRR	TCCGGCTTCAAATCCGGATTGC	This study
ihtB	FAMRTihtP	TCGTCGGAACCGTCGAGTCCGATCCCTCT	This study
	RTihtF	ACCGAGCATGCTGCCAATGA	This study
	RTihtR	CCGGTTTCTTTCAGTTCGGCAAT	This study
tlr	FAMRTtlrP	CTCTTCGGCGGTAGCTACCAGACCATGGCCGGATC	This study
	RTtlrF	AACATCATCACCCACAAATCCAAGGAC	This study
	RTtlrR	AAGCGGCCACCCAAGTTGAA	This study
hbp35	FAMRTHBP35P	TTGGTGATGGTACAGCCCCTGCAGAGGT	This study
	RTHBP35F	CTTTTTCAGTTTGCCGTATGGAGA	This study
	RTHBP35R	GCTGTACATACATCTTCCGGTGT	This study
kgp	KgpTMF	AGTAGGAACGACAAACGCCTCTA	This study
	KgpTMR	AGTGTCACCAACCAAAGCCA	This study
	fKgpBH1	AGACCGGAGCAGCACTAGCTGCCAATCCA	This study
16S	H16sPrlBRQ	CCGCCACTGAACTCAAGCCCGGCA	Gao et al [12]
	16sTMF	TCGGTAAGTCAGCGGTGAAAC	Gao et al [12]
	16sTMR	GCAAGCTGCCTTCGCAATC	Gao et al [12]

Supplementary methods

Protein purification for HusA and mutants

His-tagged HusA/mutant proteins were purified through Ni-NTA resin and the His-tag was cleaved using the Thrombin CleanCleaveTM Kit (Sigma-Aldrich, St Louis, MO). Note that the cleaved recombinant HusA comprises residues 24–218 with additional N-terminal Met-Gly di-peptide and additional C-terminal Leu-Val-Pro-Arg peptide remaining from the C-terminal engineered thrombin cleavage site. Detagged HusA and mutants were subjected to gel filtration chromatography using a Superdex-200 HiLoad 16/60 column coupled to an ÄKTA purifier system (GE Life Sciences) in 50 mM Tris, 150 mM NaCl, pH 8. The different steps in the protein purification process were checked with SDS-PAGE. The fractions containing pure HusA and mutants were stored at -80°C until use.

For NMR studies, [U-^{15}N]HusA and [U-^{13}C, ^{15}N]HusA were produced in shaker flasks using the method of Cai et al. [13] whereby initial cell mass was produced by growth in LB broth with natural abundance isotopes that was replaced, prior to protein induction, by defined medium with ^{15}N and ^{13}C isotope-labelled nutrients. The base defined medium was DM-4 [14] supplemented with trace metals and thiamine to the concentrations specified by Cai *et al.* [13] and 3 g/L D[U-^{13}C]glucose and 1.2 g/L $^{15}NH_4Cl$. Following Ni-affinity chromatography and thrombin cleavage and removal of the His-tag, isotopically labelled HusA was dialysed against 20 mM Tris, pH 7.8 at 4°C and applied to an anion exchange column (Uno-Q, BioRad) developed with a gradient 0–0.5 M NaCl over 12 column volumes. Peak HusA protein fractions were dialysed against 10 mM sodium phosphate buffer, pH 6.9.

NMR assignments and structure calculation

^{1}H 1D NMR spectra were recorded at 600 or 800 MHz using the Bruker pulse sequence p3919gp with 64 scans unless otherwise stated. Backbone ^{15}N, H^N, ^{13}C′, ^{13}Cα, Hα, ^{13}Cβ, Hβ1/β2 resonances were obtained from HNCA, HNCACB, CBCA(CO)NH, HNCO and HBHA(CO)NH experiments recorded at 298 K. Remaining aliphatic side chain ^{13}C and ^{1}H assignments were made from CC(CO)NH, HCCCONH and HCCH-TOCSY experiments recorded at 298 K and from CCH-TOCSY and HCCH-TOCSY experiments recorded at 308 K. Aromatic side chain ^{1}H and ^{13}C assignments were obtained from HBCBCGCDHDCEHE and HBCBCGCDHD experiments together with a ^{1}H,^{13}C-HSQC experiment recorded with a transmitter offset of 124 ppm, and from 2D ^{1}H COSY, TOCSY and NOESY spectra recorded on unlabelled HusA in 100% D_2O. Additional aromatic ^{1}H and ^{13}C assignments were made on the basis of cross-peaks in aromatic ^{13}C-NOESY spectra. Nε1/Hε1 resonances from three Trp side chains were assigned from cross peaks in ^{15}N-NOESY and aromatic ^{13}C-NOESY experiments. Assignments for Tyr69 and Tyr70 were confirmed from spectra recorded

on two Tyr→Phe point mutants. Side chain ^{15}Nδ1, ^{15}Nε2, Hδ2, Hε1 assignments for the single His199 residue were obtained according to Pelton et al. [15] from relative peak intensities in a ^{1}H,^{15}N-HSQC spectrum recorded with ^{15}N offset of 205 ppm and d24 = 1/4J = 11.4 ms. Stereospecific 13Cδ1/δ2 assignments for leucines 39, 50, 97, 103, 107, 113, 143, 145, 162, 163, 208, 211, 219 and 13Cγ1/γ2 and assignments for valines 31, 36, 42, 60, 124, 143, 184, 192, 220 were made on the basis of one-bond ^{13}C–^{13}C coupling of the *pro*-R methyl group (Cδ1) in ^{1}H,^{15}N-HSQC spectra recorded from a sample of [*U*-^{13}C, 10%] rHusA, as previously described [16]. Slowly exchanging side chain hydroxyl protons were assigned for Tyr69 from a 2D ^{1}H-NOESY experiment, and for Ser108 and Thr165 from a ^{15}N-NOESY experiment. Side chain amine resonances for Gln and Asn were assigned from a ^{15}N-NOESY spectrum. Arg Nε/Hε resonances were assigned for Arg114, Arg121, Arg153 from ^{15}N-HSQC and ^{15}N-NOESY spectra recorded with different ^{15}N spectral widths. A *cis*-peptidyl bond at Ser193–Pro194 was assigned based on δ(^{13}Cβ)–δ(^{13}Cγ) = 10.4 ppm [17]. Cβ chemical shifts for the five Cys residues in rHusA—Cys74 (28.3 ppm), Cys90 (27.0 ppm), Cys112 (27.0 ppm), Cys186 (27.0 ppm), Cys212 (28.1 ppm)—indicated that all were in the reduced state based on published data for proteins with known configuration [18] and theoretical considerations [19]. ^{1}H chemical shift assignments were 96% complete as measured by CYANA.

For structure calculations, 3D ^{13}C-NOESY-HSQCs (separate experiments for aliphatic and aromatic carbon resonances) and ^{15}N-NOESY-HSQC spectra were recorded at 308 K on 1–1.5 mM rHusA samples for 2–3 days each in both 100% D_2O and 93% H_2O/7% D_2O. 2D ^{1}H NOESY spectra were also recorded at 308 K in both 100% D_2O and 93% H_2O/7% D_2O. NOEs were calibrated and assigned in an automated fashion using the NOEASSIGN macro within CYANA 3 [20]. Backbone dihedral restraints were obtained from chemical shift information using TALOS+ [21]. Final structure calculations in a shell of explicit water molecules were performed using XPLOR-NIH version 2.45 [22,23] using PYTHON control scripts from PONDEROSA-C/S [24].

^{15}N relaxation measurements. Relaxation experiments were recorded at 298 K (at 600 MHz) and 308 K (at 800 MHz) on 400 μM [*U*-^{15}N]rHusA in 10 mM sodium phosphate buffer, pH 6.8. ^{15}N T_1 and T_2 and heteronuclear NOE values were measured using pulse sequences modified from Bruker pulse programs hsqct1etf3gpsi3d and hsqct2etf3gpsi3d by Dr Paul Gooley (University of Melbourne) to reduce phase error and improve water suppression. To obtain ^{15}N T_1 measurements at 298 K, relaxation delays of 12, 60, 80, 150, 200, 300 (triplicate), 600, 1000, 1400, 2000, 2600 and 5000 ms were used. For T_2 measurements at 298 K, delays of 17, 34, 102, 136, 153, 170, 221, 255 ms were used. For ^{15}N T_1 measurements at 308 K, delays of 100, 200, 400, 500, 800, 1000, 1200, 1500, 1800 and 2000 ms were used. For T_2 measurements at 308 K, delays of 16, 32, 64, 80, 112, 144, 160, 176, 224, 256 ms were used. To calculate T_1 and T_2, a two-parameter exponential decay model was fit to the data using a PYTHON extension of SPARKY; errors are the standard deviations for re-fitting *T* after

adding Gaussian noise with mean 0 and variance equal to the mean square deviation of the measured peak heights from the original best fit. ^{15}N heteronuclear NOE values were determined from the ratio of peak heights in a pair of interleaved ^{15}N-HSQC spectra recorded with or without a 5 s proton saturation period [25]; the given errors were propagated from uncertainties in peak height measurements, which were calculated as the r.m.s. noise in each processed spectrum.

Correction of inner filter effect and curve fitting in tryptophan fluorescence quenching assays

The concentration of NATA was chosen such that the raw fluorescence intensity was similar to the 10 uM concentrations of HusA and mutants used. The same molarity titrants of porphyrin were titrated into NATA as were added into HusA in the control reactions. Fluorescence intensity was scaled with respect to the starting florescence and corrected for the inner filter effect. Fluorescence at 335 nm was used to fit binding curves.

The correction method is based on Fonin AV., et al.[26], noting that a Cary Eclipse spectrofluorimeter was used.

Control titrations of porphyrins into 6 μM NATA, a fluorophore that does not bind haem, were performed to measure the contribution to the decrease in fluorescence due to the inner filter effect and this effect was scaled to the starting fluorescence values as fractional changes. The measured fluorescence at each titration point for each of the porphyrin titrations into protein was then adjusted for this contribution. Fluorescence at 335 nm was used to fit binding curves. Association constant, Ka, was fitted using the following equation in Origin, assuming a single binding site. A dissociation constant, Kd, was calculated by taking the inverse of the Ka.

$$Xobs = X_A \times f_A + X_{AB} \times f_{AB} \quad (1)$$

where X_A is the corrected fluorescence, $f_A=1-f_{AB}$, $f_{AB}=x/(Bt)$, x=(-b-sqrt(b2-4×c))/2, b=-(1/Ka+At+Bt), c=Bt×At, Bt=total concentration of protein, At=total concentration of ligand.

To aid comparison of the different titrations, the corrected fluorescence was scaled to the starting fluorescence in the absence of porphyrins (i.e. all fluorescence curves have a starting value of one after scaling).

Molecular docking

Because different conformations of the porphyrin ring (e.g., ruffling, saddling or doming distortions of the macrocycle) could not be sampled in the docking, we repeated docking for each ligand with starting coordinates that had different ring distortions. The following ring conformers were used to represent haem: HEM ligand model coordinates from pdb 1mbo; HEM ligand model coordinates from pdb 3ia3; idealised coordinates for haem calculated by the program CORINA (obtained through PDB ligand expo). The following ring conformers were used for PPIX: PP9 ligand coordinates from pdb 1hrs; PPIX PubChem CID 4971. The following ring conformers were used for DPIX: DE9 ligand coordinates from pdb 4xkb; idealised coordinates for DPIX calculated by CORINA; idealised coordinates from PubChem CID 67973. For coproporphyrin III, 3D coordinates were generated from PubChem CID 114935. For coproporphyrinogen III, coordinates from pdb 1r3t and idealized coordinates generated by CORINA were used. For uroprophyrinogen III, coordinates from pdb 2ybq and idealized coordinates generated by CORINA were used. For the P8 compound, 3D coordinates were generated using the Universal Force Field (uff) [27] or mmff94 force field [28]. The number of rotatable bonds were as follows: haem (8 active torsions); PPIX (8 active torsions); DPIX (6 active torsions); copro-porphyrin/-porphyrinogen III (12 active torsions); uro-porphyrin/-porphyrinogen III (20 active torsions). For the P8 DPIX-lysine-metronidazole compound there were 19 active torsions and two amide bonds; the amide bond linking pyrrole D propionate to the Nε-Boc-L-lysine was fixed in the *trans* conformation, whereas docking was performed with the amide bond linking the metronidazole group to the central Nε-Boc-L-lysine in either the cis or trans conformations. This did not prevent the porphyrin group adopting similar docking sites and so the results for these were pooled. Initial docking for each ligand was performed against the whole surface of HusA (~10^5 $Å^3$). Docking was then performed in a restricted volume surrounding the docking site (17576 $Å^3$) without protein side chain flexibility and the docking results were clustered into four families based on the position of the porphine ring. Docking was then performed with side chain flexibility for residues surrounding the docking site; polar (S119, Q127, Q160) and non-polar side chains (F80, L122, L123, W130 and Y164) were allowed to adopt different rotamers during docking. Docked ligands clustered into the same groups as found for docking to the rigid receptor, although the energies were more favourable by ~1.5 kcal/mol due to larger favourable Lennard Jones terms (better receptor-ligand complementarity). For example, reorientation of Y164 or F80 side chains to make face stacking interactions with the pyrrole rings.

Overall the small ring distortions in haem, PPIX and DPIX ligands did not prevent these ligands adopting the same (or similar) sets of poses and hence the results for all ring conformers over each ligand were pooled in the analysis. The non-planar coproporphyrinogen III and uroporphyrinogen III docked against the rigid receptor with lower energies (-8.4±0.4 kcal/mol and -7.1±0.5 kcal/mol, respectively) compared to haem, PPIX and DPIX (~13 kcal/mol), and were not analysed further.

Small angle X-ray scattering SAXS

Supplementary Table 2 summarises the SAXS data acquisition, analysis and results along with details of the software used as per recommended guidelines [29]. Briefly, data were reduced to I(q) versus q using the software scatterBrain. The amplitude of the momentum transfer is $I(q) = 4\pi\sin\theta/\lambda$ where 2θ is the angle between the incident and scattered X-rays and λ the wavelength. Purified apo-HusA samples were prepared by gel filtration chromatography using a Superdex-200 HiLoad 16/60 column coupled to an ÄKTA purifier system in standard buffer (50 mM Tris, 150 mM NaCl, pH 8). The HusA protein sequence includes five Cys residues, and therefore, apo-HusA samples were prepared by dialysis in standard and reduced buffer (standard buffer with 2 mM TCEP) at room temperature. For both samples, solvent scattering blanks were recorded on the end-point dialysates. The HusA:haem complex sample was prepared by adding freshly prepared haemin at a molar ratio of 1:1 to apo-HusA after dialysis in standard buffer. Given the K_d estimate of 7.3 μM for the HusA:haemin complex, it follows that, at ~140 μM HusA and haemin (i.e., 3 mg/mL of HusA), there should be minimal free haem and thus the standard buffer dialysate served as the solvent blank. Data were analysed using the ATSAS program package [8], with the specific programs used listed in Supplementary Table 2. Guinier parameters show no significant concentration dependence for apo-HusA recorded under reduced buffer, these data were therefore merged to improve statistics. The small concentration effects for data from samples in standard buffer were minimised by using the lowest concentration data for further analysis. Molecular weight estimates were calculated from Guinier I(0) values using the method of Orthaber *et. al.* [30] to yield estimates that are higher than expected for a purely monomeric sample (14−17% for HusA and 29% for HusA:haem). Making the most conservative assumption that all self-associations giving rise to this overestimate are dimeric, this result means that all samples are at least ~95% monomeric. P(r) transforms of each data set gave a bell-shaped profile consistent with the folded globular protein and a tail to high r values, likely arising from flexibility at the N and C-termini and the small amount of aggregate in the sample (Supplementary Fig 5D). Values for contrast and partial specific volumes were determined using the program MULCh [7]. Size-exclusion chromatography (SEC)-SAXS was not an option at the Australian Synchrotron for this system. SEC runs carried out in-house on a SEC-multi angle laser light scattering (MALLS) system with HusA:haem as well as HusA:haem analogues at a range of molar ratios and under several buffer conditions resulted in the haem/analogue being stripped from the complex during the sizing run. The haem/analogue was observed to have immobilised at the top of the SEC column and could not be removed with buffer. This was accompanied by increased column pressure and deteriorating column performance.

Reverse ferrochelatase activity assay

The reverse ferrochelatase activity assay was conducted to explore the ability of HusA removing iron from the haem molecule using the modified protocol described by Leob [31]. Briefly, the reaction mixture consisted of 50 mM Tris, pH 8, 0.5 mM DTT (Sigma-Aldrich), 2.5 μM haemin, 0.24 mM ferrozine (Sigma-Aldrich), 5.0 μM NADH (Sigma-Aldrich), 1% Tween 80 (Sigma-Aldrich), and approximately 0.15 mg protein in a volume of 0.83 mL. The protein concentration was determined by Bradford assay. The mixture was incubated at 37°C for 90 min. All reagents were prepared freshly. The mixtures were centrifuged at 8,000 × g for 5 min at the end of incubation. The supernatants were applied to measure the porphyrin fluorescence using SpectraMax i3 with excitation at 410 nm. The emission was recorded from 600 nm to 650 nm. The mixture without protein was used as blank.

References

1. Holm, L. & Rosenstrom, P. Dali server: conservation mapping in 3D. *Nucleic Acids Res* **38**, W545-9 (2010).
2. Moseley, H.N., Sahota, G. & Montelione, G.T. Assignment validation software suite for the evaluation and presentation of protein resonance assignment data. *J Biomol NMR* **28**, 341-55 (2004).
3. Bhattacharya, A., Tejero, R. & Montelione, G.T. Evaluating protein structures determined by structural genomics consortia. *Proteins* **66**, 778-95 (2007).
4. Kirby, N.M. *et al.* A low-background-intensity focusing small-angle X-ray scattering undulator beamline. *Journal of Applied Crystallography* **46**, 1670-1680 (2013).
5. Petoukhov, M.V. *et al.* New developments in the ATSAS program package for small-angle scattering data analysis. *J Appl Crystallogr* **45**, 342-350 (2012).
6. Gasteiger, E., Hoogland, C., Gattiker, A., Duvaud, S., Wilkins, M. R., Appel, R. D. & Bairoch, A. *Proteomics Protocols Handbook*, 571-607 (Humana Press., Totowa, 2005).
7. Whitten, A.E., Cai, S.Z. & Trewhella, J. MULCh: modules for the analysis of small-angle neutron contrast variation data from biomolecular assemblies. *Journal of Applied Crystallography* **41**, 222-226 (2008).
8. Franke, D. *et al.* ATSAS 2.8: a comprehensive data analysis suite for small-angle scattering from macromolecular solutions. *J Appl Crystallogr* **50**, 1212-1225 (2017).
9. Franke, D. & Svergun, D.I. DAMMIF, a program for rapid ab-initio shape determination in small-angle scattering. *J Appl Crystallogr* **42**, 342-346 (2009).
10. Svergun, D.I. Restoring low resolution structure of biological macromolecules from solution scattering using simulated annealing (vol 76, pg 2879, 1999). *Biophysical Journal* **77**, 2896-2896 (1999).
11. Svergun, D., Barberato, C. & Koch, M.H.J. CRYSOL - A program to evaluate x-ray solution scattering of biological macromolecules from atomic coordinates. *Journal of Applied Crystallography* **28**, 768-773 (1995).
12. Gao, J.L., Nguyen, K.A. & Hunter, N. Characterization of a hemophore-like protein from Porphyromonas gingivalis. *J Biol Chem* **285**, 40028-38 (2010).
13. Cai, M. *et al.* An efficient and cost-effective isotope labeling protocol for proteins expressed in Escherichia coli. *J Biomol NMR* **11**, 97-102 (1998).
14. Looker, D., Mathews, A.J., Neway, J.O. & Stetler, G.L. Expression of recombinant human hemoglobin in Escherichia coli. *Methods Enzymol* **231**, 364-74 (1994).
15. Pelton, J.G., Torchia, D.A., Meadow, N.D. & Roseman, S. Tautomeric states of the active-site histidines of phosphorylated and unphosphorylated IIIGlc, a signal-transducing protein from Escherichia coli, using two-dimensional heteronuclear NMR techniques. *Protein Sci* **2**, 543-58 (1993).
16. Neri, D., Szyperski, T., Otting, G., Senn, H. & Wuthrich, K. Stereospecific nuclear magnetic resonance assignments of the methyl groups of valine and leucine in the DNA-binding domain of the 434 repressor by biosynthetically directed fractional 13C labeling. *Biochemistry* **28**, 7510-6 (1989).
17. Schubert, M., Labudde, D., Oschkinat, H. & Schmieder, P. A software tool for the prediction of Xaa-Pro peptide bond conformations in proteins based on 13C chemical shift statistics. *J Biomol NMR* **24**, 149-54 (2002).
18. Sharma, D. & Rajarathnam, K. 13C NMR chemical shifts can predict disulfide bond formation. *J Biomol NMR* **18**, 165-71 (2000).

19. Martin, O.A., Villegas, M.E., Vila, J.A. & Scheraga, H.A. Analysis of 13Calpha and 13Cbeta chemical shifts of cysteine and cystine residues in proteins: a quantum chemical approach. *J Biomol NMR* **46**, 217-25 (2010).
20. Guntert, P. & Buchner, L. Combined automated NOE assignment and structure calculation with CYANA. *J Biomol NMR* **62**, 453-71 (2015).
21. Shen, Y., Delaglio, F., Cornilescu, G. & Bax, A. TALOS+: a hybrid method for predicting protein backbone torsion angles from NMR chemical shifts. *J Biomol NMR* **44**, 213-23 (2009).
22. Schwieters, C.D., Kuszewski, J.J., Tjandra, N. & Clore, G.M. The Xplor-NIH NMR molecular structure determination package. *J Magn Reson* **160**, 65-73 (2003).
23. Schwieters, C.D., Kuszewski, J.J. & Clore, G.M. Using Xplor-NIH for NMR molecular structure determination. *Progress in Nuclear Magnetic Resonance Spectroscopy* **48**, 47-62 (2006).
24. Lee, W., Stark, J.L. & Markley, J.L. PONDEROSA-C/S: client-server based software package for automated protein 3D structure determination. *Journal of Biomolecular Nmr* **60**, 73-75 (2014).
25. Farrow, N.A. *et al.* Backbone Dynamics of a Free and a Phosphopeptide-Complexed Src Homology-2 Domain Studied by N-15 Nmr Relaxation. *Biochemistry* **33**, 5984-6003 (1994).
26. Fonin, A.V., Sulatskaya, A.I., Kuznetsova, I.M. & Turoverov, K.K. Fluorescence of dyes in solutions with high absorbance. Inner filter effect correction. *PLoS One* **9**, e103878 (2014).
27. Rappe, A.K., Casewit, C.J., Colwell, K.S., Goddard, W.A. & Skiff, W.M. Uff, a Full Periodic-Table Force-Field for Molecular Mechanics and Molecular-Dynamics Simulations. *Journal of the American Chemical Society* **114**, 10024-10035 (1992).
28. Halgren, T.A. Merck molecular force field .2. MMFF94 van der Waals and electrostatic parameters for intermolecular interactions. *Journal of Computational Chemistry* **17**, 520-552 (1996).
29. Trewhella, J. *et al.* 2017 publication guidelines for structural modelling of small-angle scattering data from biomolecules in solution: an update. *Acta Crystallographica Section D-Structural Biology* **73**, 710-728 (2017).
30. Orthaber, D., Bergmann, A. & Glatter, O. SAXS experiments on absolute scale with Kratky systems using water as a secondary standard. *Journal of Applied Crystallography* **33**, 218-225 (2000).
31. Loeb, M.R. Ferrochelatase activity and protoporphyrin IX utilization in Haemophilus influenzae. *J Bacteriol* **177**, 3613-5 (1995).
